# Discovery of two new isoforms of the human DUT gene

**DOI:** 10.1038/s41598-023-32970-1

**Published:** 2023-05-12

**Authors:** Gergely Attila Rácz, Nikolett Nagy, György Várady, József Tóvári, Ágota Apáti, Beáta G. Vértessy

**Affiliations:** 1 Department of Applied Biotechnology and Food Sciences, Faculty of Chemical Technology and Biotechnology, BME Budapest University of Technology and Economics, Műegyetem Rkp. 3., Budapest, 1111 Hungary; 2 Institute of Enzymology, Research Centre for Natural Sciences, ELKH Eötvös Loránd Research Network, Budapest, Hungary; 3 Doctoral School of Biology, Institute of Biology, ELTE Eötvös Loránd University, 1117 Budapest Pázmány Péter Sétány 1/C, Budapest, Hungary; 4 Department of Experimental Pharmacology, National Institute of Oncology, Ráth Gy. U. 7-9, Budapest, 1122 Hungary

**Keywords:** DNA repair enzymes, Isoenzymes, Reverse transcription polymerase chain reaction, Cancer models, Nucleotide-binding proteins, Biochemistry, Cancer, Cell biology, Molecular biology

## Abstract

In human cells two dUTPase isoforms have been described: one nuclear (DUT-N) and one mitochondrial (DUT-M), with cognate localization signals. In contrast, here we identified two additional isoforms; DUT-3 without any localization signal and DUT-4 with the same nuclear localization signal as DUT-N. Based on an RT-qPCR method for simultaneous isoform-specific quantification we analysed the relative expression patterns in 20 human cell lines of highly different origins. We found that the DUT-N isoform is expressed by far at the highest level, followed by the DUT-M and the DUT-3 isoform. A strong correlation between expression levels of DUT-M and DUT-3 suggests that these two isoforms may share the same promoter. We analysed the effect of serum starvation on the expression of dUTPase isoforms compared to non-treated cells and found that the mRNA levels of DUT-N decreased in A-549 and MDA-MB-231 cells, but not in HeLa cells. Surprisingly, upon serum starvation DUT-M and DUT-3 showed a significant increase in the expression, while the expression level of the DUT-4 isoform did not show any changes. Taken together our results indicate that the cellular dUTPase supply may also be provided in the cytoplasm and starvation stress induced expression changes are cell line dependent.

## Introduction

Polymerases cannot distinguish between dUTP and dTTP as they differ in only one methyl group, therefore maintaining the appropriate dUTP/dTTP ratio is of utmost importance^1^. The enzyme dUTPase is responsible for preserving the integrity of the genome by catalysing the hydrolysis of dUTP into dUMP and pyrophosphate, thus eliminating dUTP from the dNTP pool^2,3^. If dUTP is available, the incorporated uracil is cleaved by the uracil-DNA glycosylases as a part of the base excision repair mechanism^4^. Elevated dUTP level can lead to thymineless cell death via the overactivation of the DNA repair process^5^, to prevent this, the activity of the dUTPase enzyme is required. The other role of the reaction catalysed by dUTPase is to produce dUMP, which is a substrate for thymidylate synthase in the de novo dTTP synthesis pathway.

To date, two isoforms of human dUTPase have been described in the literature, which localize to the nucleus and to the mitochondrion, respectively, presumably to ensure dUTP pool regulation in these two DNA containing organelles^6^. The two isoforms are encoded by the DUT gene and generated by alternative promoter usage coupled with alternative splicing^7,8^. The two isoforms differ only in the first exon, as the mitochondrial isoform (DUT-M) contains a mitochondrial targeting sequence, while the nuclear isoform (DUT-N) contains a nuclear localization signal^7,9,10^. The two isoforms of dUTPase were described by Ladner et al. with northern and western blot analysis at the mRNA and protein levels, respectively^7^. The RNA expression levels of the isoforms in 34Lu human lung fibroblast cells were also investigated under serum starvation, which forces cells to exit from the cell cycle and enter into a resting state. According to Ladner et al., this transition leads to a drastic decrease in the expression of the DUT-N dUTPase isoform, with no change in the level of the DUT-M isoform.

Recent high-throughput sequencing studies predicted the putative presence of two additional isoforms in humans, termed as DUT-3 (UniProt ID: A0A0C4DGL3, NCBI RefSeq ID: NM_001025249.1) and DUT-4 (UniProt ID: H0YNW5, NCBI RefSeq ID: NM_001330286.2) in the present work. Sequence databases suggested that the third suggested isoform (DUT-3) does not contain any localisation signal, thus it is most probably retained in the cytosol. The upstream part of the 5’ UTR region of DUT-3 is identical to that of DUT-M, however, the mitochondrial leader sequence—present in the first exon of the DUT-M isoform—is absent from the DUT-3 isoform. The fourth suggested isoform (DUT-4) was predicted to closely resemble the DUT-N isoform, only differing in a few amino acids at the N-terminus. The first exon of this isoform is located upstream from the other isoforms’ in the genome, therefore, its expression may be driven by an alternative promoter for a potential altered regulation of this isoform. No data has yet been reported on the physiological expression levels or role(s) of these two novel human dUTPase isoforms.

Here we present gene expression data of the four isoforms of human dUTPase in various cancer and normal human cell lines in order to gain insight into the physiological role of the new isoforms. RT-qPCR was our method of choice to identify the two novel isoforms and quantify the mRNA expression of all four isoforms separately in various cell lines. The advantages of RT-qPCR include its excellent specificity, wide linear dynamic range, outstanding sensitivity and reproducibility^11–15^. To develop a reliable RT-qPCR method for gene expression analysis, thorough optimisation and the use of appropriate reference genes are required^16–18^. In a previous article we identified novel reference genes in the same human normal and cancer cell lines that we use in this study^19^. Furthermore, we used the same approach and performed the same optimisation steps to develop an RT-qPCR method for the analysis of the gene expression of the dUTPase isoforms.

## Results and discussion

### Overview of the isoform-specific determination of dUTPase gene expression

Our aim was to determine the mRNA expression level of the dUTPase isoforms specifically. First, we investigated the Ensemble, the UniProt, and the NCBI Reference Sequences (RefSeq) databases and also took into account the Consensus CDS (CCDS) project. In the Ensemble database, 8 protein coding and 3 non-protein coding isoforms are present, out of which 9 have UniProt identifiers (P33316 [DUT-M], P33316-2 [DUT-N], A0A0C4DGL3 [DUT-3], H0YNW5 [DUT-4], H0YNJ9, H0YKC5, H0YMP1, H0YMM5, H0YKI0). We performed multiple protein sequence alignment with the online tool Clustal Omega using the default settings. Supplementary Fig. 1. shows the results of the alignment. The enzyme dUTPase has five conserved motifs among eukaryotes, prokaryotes, DNA viruses and retroviruses^2^. Out of the 9 isoforms described in the UniProt database, only 4 contain all conserved motifs, including the DUT-N and DUT-M well-known isoforms, and two novel isoforms, termed as DUT-3 and DUT-4 in the present article. Furthermore, only these 4 isoforms are present in the NCBI RefSeq database (NM_001025248.2 [DUT-M], NM_001948.4 [DUT-N], NM_001025249.1 [DUT-3] and NM_001330286.2 [DUT-4]) and have CCDS identifiers (CCDS32231 [DUT-M], CCDS45255 [DUT-N], CCDS45256 [DUT-3] and CCDS81879 [DUT-4]). The CCDS database contains sequences that are consistently annotated and of high quality. Moreover, we investigated the human dUTPase isoforms in the PeptideAtlas database^20^. Besides the canonical DUT-N and DUT-M isoforms, the only proteins with 100% peptide coverage are the DUT-3 and DUT-4. Peptide coverage of the other hypothetical isoforms incomplete, questioning the cellular existence of these proteins. In summary, we aimed at investigating only functional four isoforms: DUT-M, DUT-N, DUT-3 and DUT-4.

**Figure 1:**
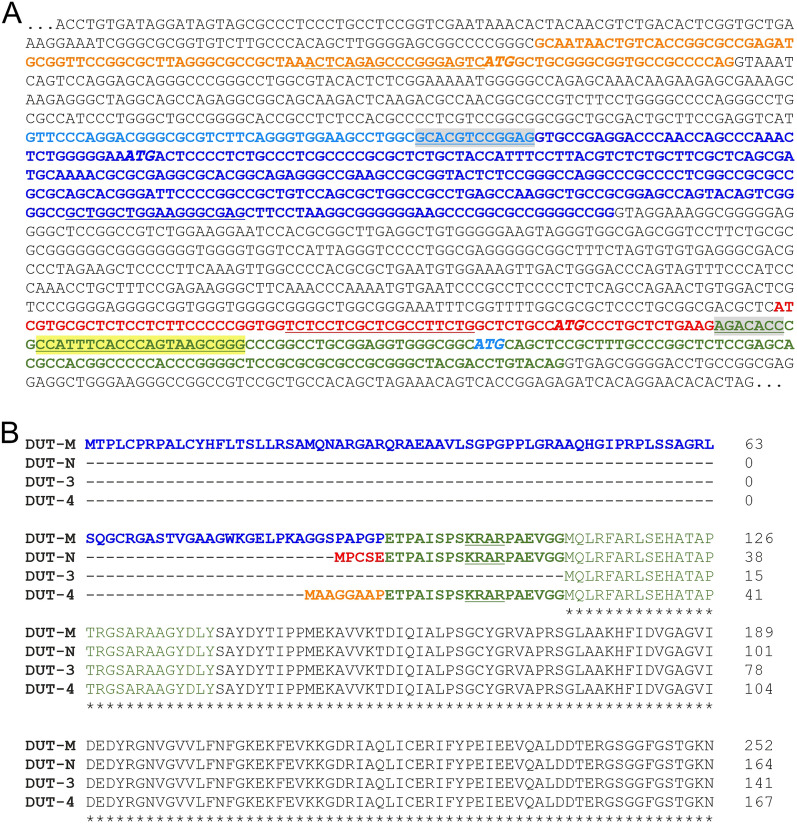
Partial genomic sequence **(A)** and the protein sequences of the dUTPase isoforms **(B). Panel **(**A**)**:** The first exon of the DUT-4 isoform is shown in orange, the first exon of the DUT-3 isoform is shown in light blue. The first exon of the DUT-M isoform contains the first exon of the DUT-3 isoform (light blue) and also contains the dark blue sequence segment, unique to the DUT-M isoform. The first exon of the DUT-N isoform is shown in red and green sequence segments, where the sequence in red is unique to the DUT-N isoform while the sequence in green is common to all isoforms. The primer sequences used in RT-qPCR are underlined. The intron-spanning forward primer designed for the DUT-3 isoform is shown in grey background. The common reverse primer is shown in yellow background. The translation initiation sites (ATG) are shown in italic shadow text coloured as the corresponding isoform (DUT-M, dark blue; DUT-N, red; DUT-3, light blue; DUT-4, orange) . (**B**) Protein sequence of the dUTPase isoforms. The peptide segment corresponding to the first exon is coloured according to the genomic sequence, the other exonic sequences are in black. The dark green coloured sequence is common for all isoforms except for DUT-3. The light green coloured sequence is common to all isoforms. The core nuclear localisation signal (KRAR) is underlined. Figure was created with Microsoft Office 2013.

Figure 1A shows the promoter region of the genomic sequence of the DUT gene. All dUTPase isoforms are generated by alternative promoter usage and alternative splicing, however, all isoforms share the same 3’ end at the mRNA level and corresponding C-terminus at the protein level. Besides determining the gene expression levels of the four dUTPase isoforms, we also aimed to determine the expression of all isoforms together (DUT-all) by designing primer pairs located in the common sequence. Detailed explanation of the primer design used for the isoform-specific determination can be found in Materials and methods. Table 1 contains key parameters of the primers used in this study. Figure 1B shows the.

**Table 1:** List of the investigated DUT isoforms and the corresponding primer parameters used in this study. Regression coefficient values were determined by performing least squares linear regression to the average Cq values of technical replicates. PCR product length is also indicated in bp (base pairs).

Gene symbol	Primer sequences (5'-3')	PCR product length (bp)	Tm of PCR products (°C)	Primer design	PCR amplification efficiency (%)	Regression Coefficient (R-squared)
DUT-M	Fw: GCTGGCTGGAAGGGCGAG Rev: CCCGCTTACTGGGTGAAATGG	85	88.0	Intron-flanking	96.6	0.99991
DUT-N	Fw: TCTCCTCGCTCGCCTTCTG Rev: CCCGCTTACTGGGTGAAATGG	73	83.5	Exonic	97.0	0.99989
DUT-3	Fw: GCACGTCCGGAGAGACACC Rev: CCCGCTTACTGGGTGAAATGG	42	79.4	Intron-spanning	99.3	0.99974
DUT-4	Fw: ACTCAGAGCCCGGGAGTC Rev: CCCGCTTACTGGGTGAAATGG	73	86.3	Intron-flanking	96.4	0.99979
DUT-all	Fw: ACTCATATGATCTCCCTTCAGCAA Rev: ATGCAAGATTTTGTACCTTGTGAAAG	109	74	Exonic	96.1	0.99994

### RNA extraction and quality control

In our previous article, we identified appropriate reference genes for normalization of relative gene expression measured with RT-qPCR^19^. In our current study we use the same RNA samples prepared previously. We prepared three biological replicate RNA samples from 20 human cancer and normal cell lines. Shortly, normal and cancer cell lines were cultured and harvested from three biological replicates. After extraction, the integrity of the RNA samples were investigated with agarose gel electrophoresis. Two distinct bands were visible on the agarose gel image, which correspond to the 18S and 28S ribosomal RNA subunits, indicating lack of degradation and genomic DNA contamination, thus an overall good quality of the prepared RNA samples. To determine the concentration and the purity of the RNA samples, NanoDrop measurements were performed. The 260/280 ratios were in the range from 2.02 to 2.11 demonstrating lack of protein contamination. The 260/280 and 260/230 absorbance ratios and the RNA yields along with the gel electrophoresis results are summarized in our previous article as supplementary material^19^.

### Optimisation of the reverse transcription reaction

**Figure 2:**
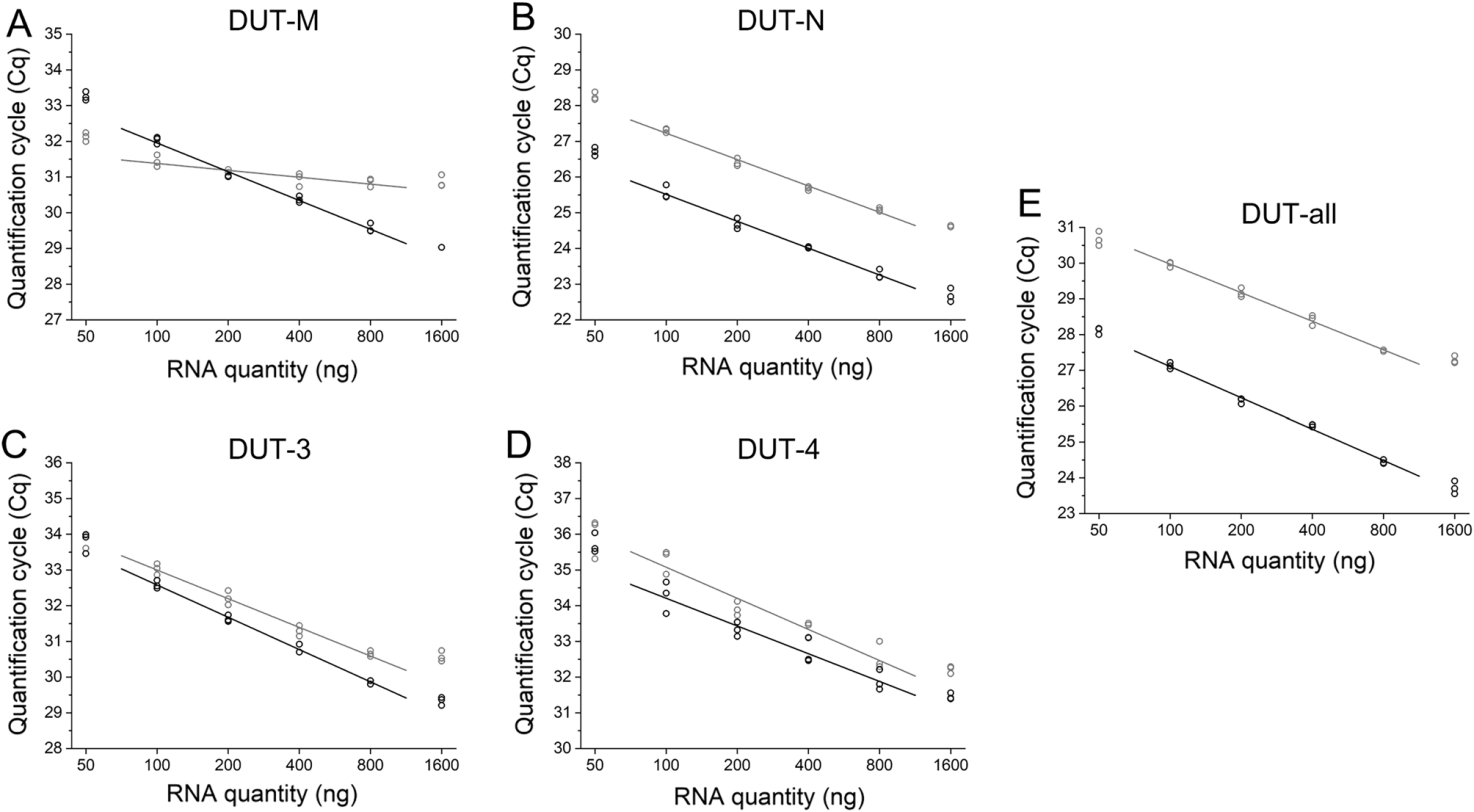
Optimization of the reverse transcription reaction. Cq values resulting from qPCR measurements of RNA dilution series are shown comparing the Maxima First Strand cDNA Synthesis Kit for RT-qPCR (black lines) and the High-Capacity cDNA Reverse Transcription Kit (grey lines). At each concentration point three technical replicates for both kits are depicted as hollow circles. Least squares linear regression was performed to the average of the technical replicates in the range of RNA amount from 100 to 800 ng per reaction. Individual graphs for (**A**) DUT-M, (**B**) DUT-N, (**C**) DUT-3, (**D**) DUT-4 and for (**E**) DUT-all were created with OriginPro 2018 (OriginLab Corp.) and the figure was assembled using CorelDRAW Graphics Suite 2020 (Corel Corporation).

The performance of the reverse transcription (RT) reaction highly depends on the reverse transcriptase enzyme, the priming strategy and the amount of the RNA in the reaction^21–23^. It is fundamental to work within the linear dynamic range of the RT reaction to gain reliable gene expression results with qPCR. The investigation of each target RNA is crucial including the targets of interest and reference genes, as well. For the reference genes used in this study, the same optimisation process discussed below was performed and the results were published in our previous article^19^. We compared two commercially available reverse transcription kits—the Maxima First Strand cDNA Synthesis Kit for RT-qPCR and the High-Capacity cDNA Reverse Transcription Kit. After preparing a series of 6 point fourfold dilutions from an RNA sample, the reverse transcription reaction was performed using each kit. The quantity of total RNA in the reaction ranged from 50 to 1600 ng. The most and least concentrated points fell out of the linear dynamic range, however, linearity was confirmed in the range between 100 and 800 ng RNA for all targets including the reference genes (Fig. 2). We performed least squares linear regression to the average of the technical replicates within the range of 100–800 ng RNA. The Maxima First Strand cDNA Synthesis Kit for RT-qPCR resulted in lower Cq values indicating better reverse transcription efficiency for all targets including the reference genes. In case of the DUT-M isoform, the slope of the linear fitted line was definitely flatter using the High-Capacity cDNA Reverse Transcription Kit, while using the Maxima First Strand cDNA Synthesis Kit for RT-qPCR, the slope of the linear fitted line was adequate. Using the former kit, the sensitivity of the determination of the DUT-M isoform would be clearly compromised. Accordingly, for further experiments the Maxima First Strand cDNA Synthesis Kit for RT-qPCR was selected and 200 ng total RNA was used.

### Determination of qPCR efficiency

For the accurate quantification of the expression of target genes, determining the qPCR efficiency for each target is crucial, moreover precise and robust qPCR methods are characterized with high efficiency^24^. Dilution series from PCR products were prepared and used as template in following qPCR reactions using three technical replicates. Using this approach a broad concentration range can be investigated, however using serial dilutions of cDNA template for determining the efficiency would take the matrix effect into consideration^25^. Therefore, the efficiency was determined also with cDNA template for the DUT-N isoform (97.7%) and the DUT-all target (100%). Comparing the two approaches we did not find a considerable difference in the efficiency values—97% for the DUT-N isoform and 96.1% for the DUT-all target using PCR products as template. The resulting Cq values were plotted against the applied dilution and least squares linear regression was performed to the average of the technical replicates (Supplementary Figure S2). The efficiency values were calculated from the steepness of the fitted line^26^. The efficiency values and the regression coefficient values are summarized in Table 1.

### Confirmation of the PCR product sequences

To verify the specificity of the PCR products regarding the four isoforms, we sent PCR products for Sanger sequencing, however as the length of all PCR products are below 90 base pairs, a nested PCR design was applied^27^. A longer PCR product was generated for each target with another reverse primer and the appropriate forward primer and these products were purified and analysed with Sanger sequencing. The sequence of the longer PCR products were identical with the corresponding sequence in public databases. These longer PCR products contain the sequence of the respective single-round PCR products. Then, the sequenced products were diluted and used as template in a second round of PCR—using the primers listed in Table 1—in addition to a human cDNA template. The identity of the two PCR products from the nested and the single-round PCR reaction was assessed by comparing the results of melting curve analysis and agarose gel electrophoresis. Since the melting curves were identical (Supplementary Figure S3) and the bands appeared in the same position on agarose gel (Supplementary Figure S4), we concluded that every PCR product is specific for the intended target.

### Reference genes used for gene expression determination

In our previous work, we investigated 12 candidate reference genes in the same human normal and cancer cell lines that we use in the present study^19^. We identified SNW1 and CNOT4 as novel candidate reference genes based on the RNA HPA cell line gene data from The Human Protein Atlas^28^. Along with widely used reference genes (ACTB, GAPDH, IPO8, PPIA, PUM1, RPL30, TBP and UBC) we also included HNRNPL and PCBP1 in our study as suggested by Jo et al.^29^. Several approaches were applied to evaluate the results such as GeNorm, NormFinder, BestKeeper and the Comparative ΔCt methods. For a reliable normalization the use of at least two reference genes is recommended to minimize experimental bias^16,30,31^. Based on our results, we suggested the use of IPO8, PUM1, HNRNPL, SNW1 and CNOT4 as stable reference genes^19^, accordingly, we used this set of genes as references for gene expression analysis of the dUTPase isoforms. For evaluating the effect of serum starvation, CNOT4, PUM1 and PCBP1 were used as reference genes.protein sequences for the dUTPase isoforms, which are coloured according to the genomic sequence.

### Gene expression analysis of the dUTPase targets

After preparing three biological replicate RNA samples from our set of human cancer and normal cell lines, the expression of the dUTPase isoforms, as well as the DUT-all target was investigated. For this purpose the total RNA quantity used in the reverse transcription reaction and the volume of cDNA amplified in the qPCR reaction were kept constant. We used the ΔΔCq method for the calculation of gene expression values applying IPO8, PUM1, HNRNPL, SNW1 and CNOT4 as reference genes. The amplification curves for the cell lines having highest and lowest relative normalized expression of the dUTPase isoforms and the DUT-all target are illustrated in Supplementary Figure S5. Based on the Cq values, the DUT-N isoform has the highest expression of the dUTPase isoforms in all cell lines investigated, followed by the DUT-M isoform and the DUT-3 isoform. The DUT-4 isoform has the lowest expression of the dUTPase isoforms, however the DUT-3 and the DUT-4 isoforms are expressed to a similar extent in some of the cell lines investigated.

**Figure 3:**
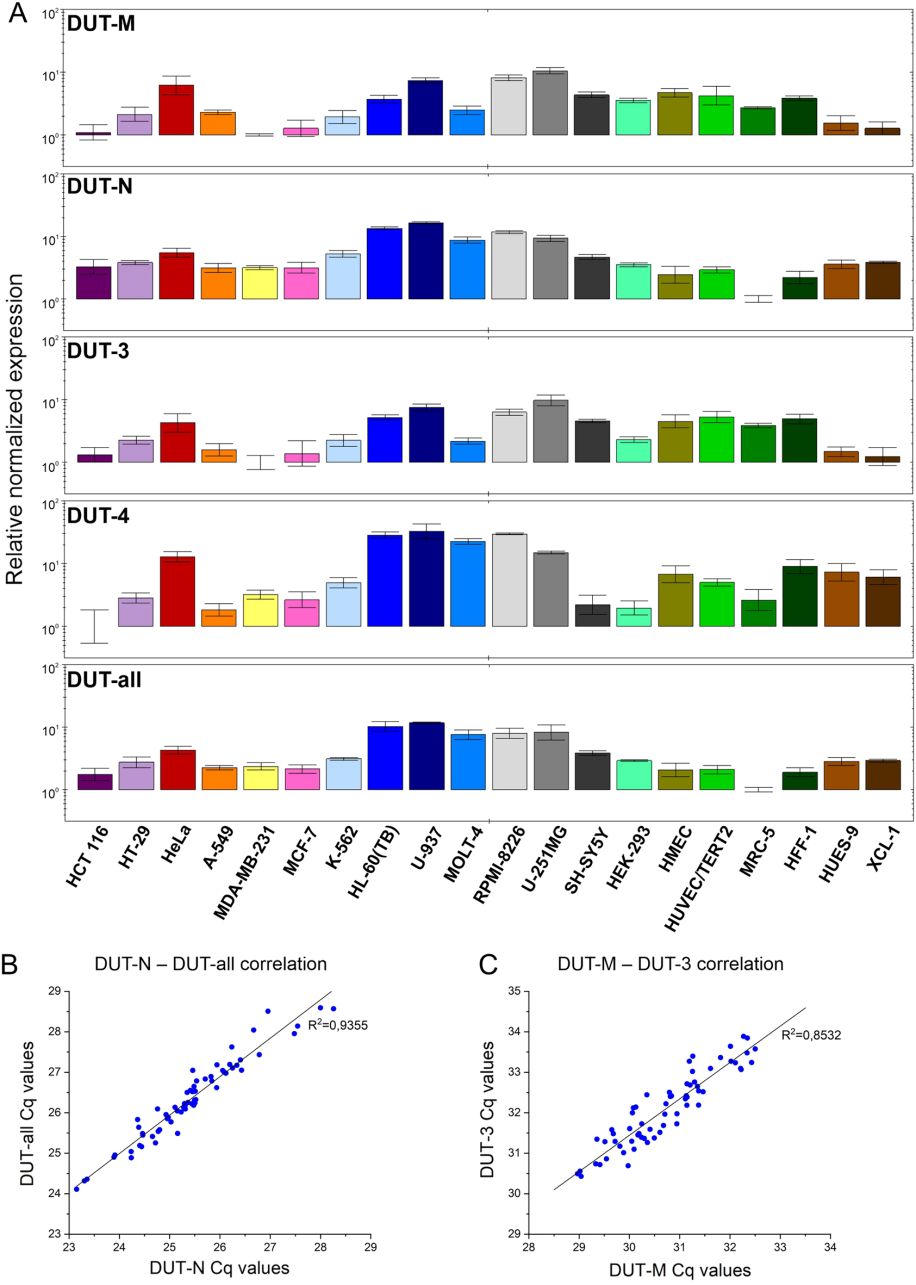
Relative normalized gene expression data for the dUTPase targets and correlation analysis of the Cq values. (**A**) In each panel, the expression value of the cell line with the lowest expression was set to 1. The scale of the y axis is logarithmic with base 10 and is shown uniformly from 1 to 100 in each panel. The error bar shows the standard deviation of three biological replicates (n = 3) for each cell line. The individual colours correspond to the colours used in Table 2. Individual graphs were created with CFX Maestro 2.0 (Bio-Rad). (**B**) Correlation analysis of the Cq values of the DUT-N with the DUT-all target and (**C**) the DUT-M with the DUT-3 target. The range displayed on the axes is constant in both graphs for comparability. Regression coefficient values were determined with least squares linear regression to all data points. Individual graphs were created with OriginPro 2018 (OriginLab Corp.) and the figure was assembled using CorelDRAW Graphics Suite 2020 (Corel Corporation).

The relative normalized expression values for each dUTPase isoform and the DUT-all target for the twenty cell lines investigated are illustrated in Fig. 3A. The relative normalized expression values along with the standard deviation are summarized in Supplementary Table S1. The relative normalized expression of the DUT-M and the DUT-3 isoforms was found to be highest in U-937, RPMI-8226 and U-251MG cell lines and lowest in MDA-MB-231 cell line. In case of the DUT-N, the DUT-4 and the DUT-all target, the relative normalized expression was considerably high in HL-60(TB), U-937, MOLT-4, RPMI-8226 and U-251MG cell lines. Comparing the human pluripotent stem cell line HUES-9 and the induced pluripotent stem cell line XCL-1, the relative normalized expression of each isoform and the DUT-all target does not differ significantly as the *p*-values were greater than or equal to 0.4 in all cases. Expression levels of the DUT-4 isoform showed the largest differences among the cell lines, with a 50-fold difference between the highest (U-937) and lowest (HCT-116) expression level.

**Table 2:** Cell lines used in this study. The colour code corresponds to the colours used in Fig. 3.

Accession (RRID)	Cell line	Colour code	Disease	Cell type	Category
CVCL_0291	HCT 116		Colon carcinoma	Intestinal epithelial cell	Adenocarcinoma
CVCL_0320	HT-29		Colon adenocarcinoma	Intestinal epithelial cell	
CVCL_0030	HeLa		Human papillomavirus-related endocervical adenocarcinoma	Epithelial cell	
CVCL_0023	A-549		Lung adenocarcinoma	Alveolar basal epithelial cell	
CVCL_0062	MDA-MB-231		Breast adenocarcinoma	Mammary gland basal B epithelial cell	
CVCL_0031	MCF-7		Invasive breast carcinoma	Mammary gland luminal A epithelial cell	
CVCL_0004	K-562		Chronic myelogenous leukemia, BCR-ABL1 positive	Highly undifferentiated myeloid cell	Leukemia
CVCL_A794	HL-60(TB)		Adult acute myeloid leukemia	Promyelocyte	
CVCL_0007	U-937		Adult acute monocytic leukemia	Monocyte	
CVCL_0013	MOLT-4		Adult T acute lymphoblastic leukemia	Precursor T-cell	
CVCL_0014	RPMI-8226		Plasma cell myeloma	B lymphocyte	Other cancer cell line
CVCL_0021	U-251MG		Astrocytoma	Astrocyte	
CVCL_0019	SH-SY5Y		Neuroblastoma	Neuron (dopaminergic/adrenergic)	
CVCL_0045	HEK293		Normal—transformed with Ad5	Adrenal precursor cell	Normal cell line
CVCL_UW69	HMEC		Normal—immortalized with TERT	Mammary epithelial cell	
CVCL_9Q53	HUVEC/TERT2		Normal—immortalized with TERT	Umbilical vascular endothelial cell	
CVCL_0440	MRC-5		Normal	Embryo lung fibroblast	
CVCL_3285	HFF-1		Normal	Foreskin fibroblast	
CVCL_0057	HUES-9		Normal	Embryonic stem cell from blastocyst	Stem cells
CVCL_WM82	XCL-1		Normal	Induced pluripotent stem cell	

To reveal potential common regulatory elements among the relative expression levels of the different isoforms, we analysed the correlation of the Cq values of every combination of two targets of the dUTPase isoforms and the DUT-all target (Supplementary Figure S6). We found correlation between the Cq values of the DUT-N isoform and the DUT-all target (Fig. 3B), moreover, between the DUT-M and the DUT-3 isoforms (Fig. 3C). Both correlations were characterized with regression coefficient values higher than 0.85 determined with least squares linear regression to all data points. In contrast, all other combinations were characterized with regression coefficient values below 0.62, indicating lack of correlation. The correlation analysis provided three important observations. First, since DUT-N has the highest expression of the dUTPase isoforms based on the amplification curves, this isoform dominates the overall expression of dUTPase, as it is reflected in the strong correlation between the DUT-N and the DUT-all expression patterns. Second, considering that both the DUT-M and the DUT-3 isoforms are transcribed from the same promoter, the correlation between these two targets was expected and this finding further strengthens the suggestion for a common promoter for DUT-M and DUT-3. Third, lack of correlation for any combinations involving the DUT-4 isoform argues for the presence of an alternative promoter that provides independent and different regulation of expression for DUT-4.

**Figure 4:**
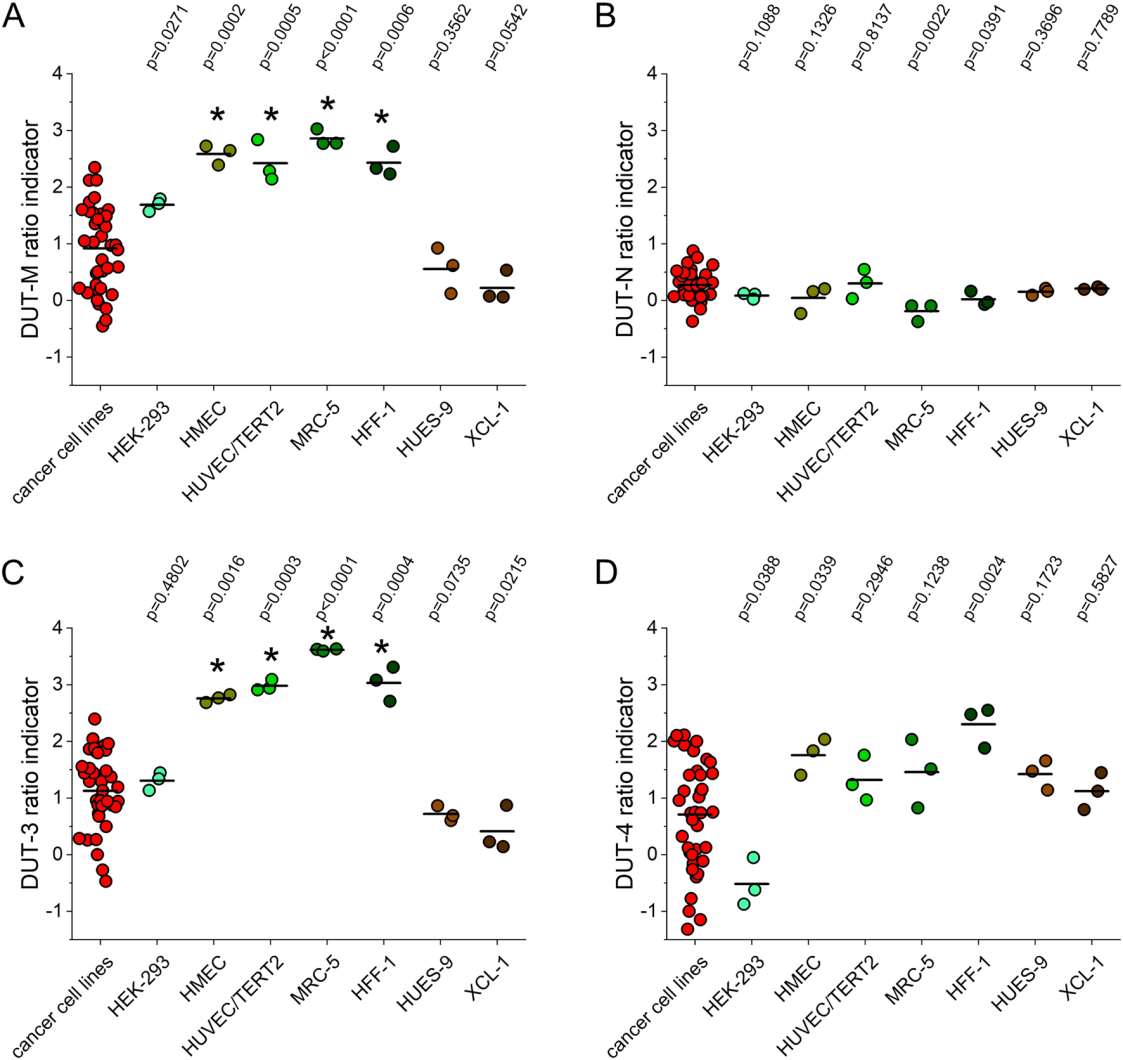
Relation of the relative normalized expression level of the different isoforms (**A**) DUT-M, (**B**) DUT-N, (**C**) DUT-3, (**D**) DUT-4 to the overall dUTPase expression measured with the DUT-all target. All cancer cell lines were included in a group which were used for pairwise comparisons to each normal cell line. Cancer cell lines are coloured red. The individual colours for the normal cell lines correspond to the colours used in Table 2. The y axis shows ratio indicators that are the ratio of the relative normalized expression value of each isoform to the overall expression value on a logarithmic scale. The mean of each group is indicated with black lines. The *p* values indicate the results of pairwise comparisons. The differences were found to be significant at *p *< 0.0018 and are indicated with asterisks (*). Individual graphs were created with OriginPro 2018 (OriginLab Corp.) and the figure was assembled using CorelDRAW Graphics Suite 2020 (Corel Corporation).

As the ratio of different isoforms expressed may be important for the proper functioning of a cell, we investigated the pattern of the dUTPase isoforms expressed compared to the overall expression in normal and cancer cell lines. We set the relative normalized expression of one biological replicate sample of MDA-MB-231 cell line to 1. We divided the relative normalized expression values of each isoform with the DUT-all relative normalized expression values, and the base 2 logarithm of the ratio was calculated to provide normal distribution. To describe the relation of the expression level of the different isoforms, we used these values termed “ratio indicators” to emphasise that the numerical values are not to be considered, only the differences observed are of interest. As the variance of the three biological replicate samples measured in different cell lines is not equal, we performed the non-parametric Kruskal–Wallis analysis followed by Conover-Iman pairwise comparisons with Bonferroni correction using XLSTAT. For each isoform the ratio indicators of normal cell lines were compared to the group of cancer cell lines. Figure 4. illustrates the results.

In case of the DUT-N and the DUT-4 isoforms, no significant differences were found. As the DUT-N has the highest expression, the relation of this isoform to the overall expression was expected to be nearly equal among the cell lines. The DUT-4 isoform has the lowest expression, but its ratio indicator also does not show significant changes, it is quite stable among the normal cell lines. However, in case of the DUT-M and DUT-3 isoforms, differences were observed among the normal cell lines. The HEK293, the HUES-9 and the XCL-1 cell lines were not found to be significantly different from the cancer cell lines group. The human pluripotent stem cell line HUES-9 and the induced pluripotent stem cell line XCL-1 showed similar, not elevated ratio indicator values arguing for the resemblance of these cell lines. The HEK293 is a partially differentiated precursor cell line transformed with Ad5, and this may be the reason for its divergence from the normal cell group. In contrast, the differentiated normal cell lines HMEC, HUVEC/TERT2, MRC-5 and HFF-1 showed significantly increased values. The ratio indicator values for the DUT-M and the DUT-3 isoforms demonstrated parallel changes further arguing for their common promoter.

### Gene expression analysis of the dUTPase targets upon serum starvation: cell-line specific differences

Serum starvation is a commonly applied treatment used in several fields of research investigating human cell lines to reveal molecular mechanisms involved in different cellular processes, metabolic pathways and effects of drug treatments^32^. Previously, the relative mRNA expression of DUT-N and DUT-M upon serum starvation was investigated in 34Lu normal human lung fibroblast cells by Ladner et al. using northern blot technique^7^. Results from this study showed that the mRNA expression of DUT-M was constitutive, however, the DUT-N mRNA expression decreased significantly upon serum starvation. It was of interest to investigate the expression of the novel two isoforms of dUTPase, as well as the DUT-N and DUT-M and also the DUT-all target under serum starvation treatment in various cell lines to decide whether the effects are cell-line dependent. Considering our set of 13 human cancer cell lines, cell cycle arrest cannot be achieved efficiently for most cell lines either because the growth of the cells is unaffected or the cell viability is heavily compromised upon serum starvation. Therefore, we selected three human cancer cell lines—HeLa, A-549 and MDA-MB-231—for which this treatment is applicable^33^. Serum starvation was applied to three biological replicate cultures for 2 days in case of MDA-MB-231 cell line and for 4 days in case of HeLa and A-549 cell lines. The cell cycle phase distribution of the treated and non-treated cell cultures was analysed with flow cytometry. Elevation of the ratio of the cells in G1 phase was observed in parallel to a decrease in the ratio of the cells in G2 and S phase upon serum starvation (Supplementary Figure S7). The ratio of the cells in G1 phase in serum starved cells was higher than 70% in every case, therefore, cell cycle arrest was achieved successfully.

**Figure 5:**
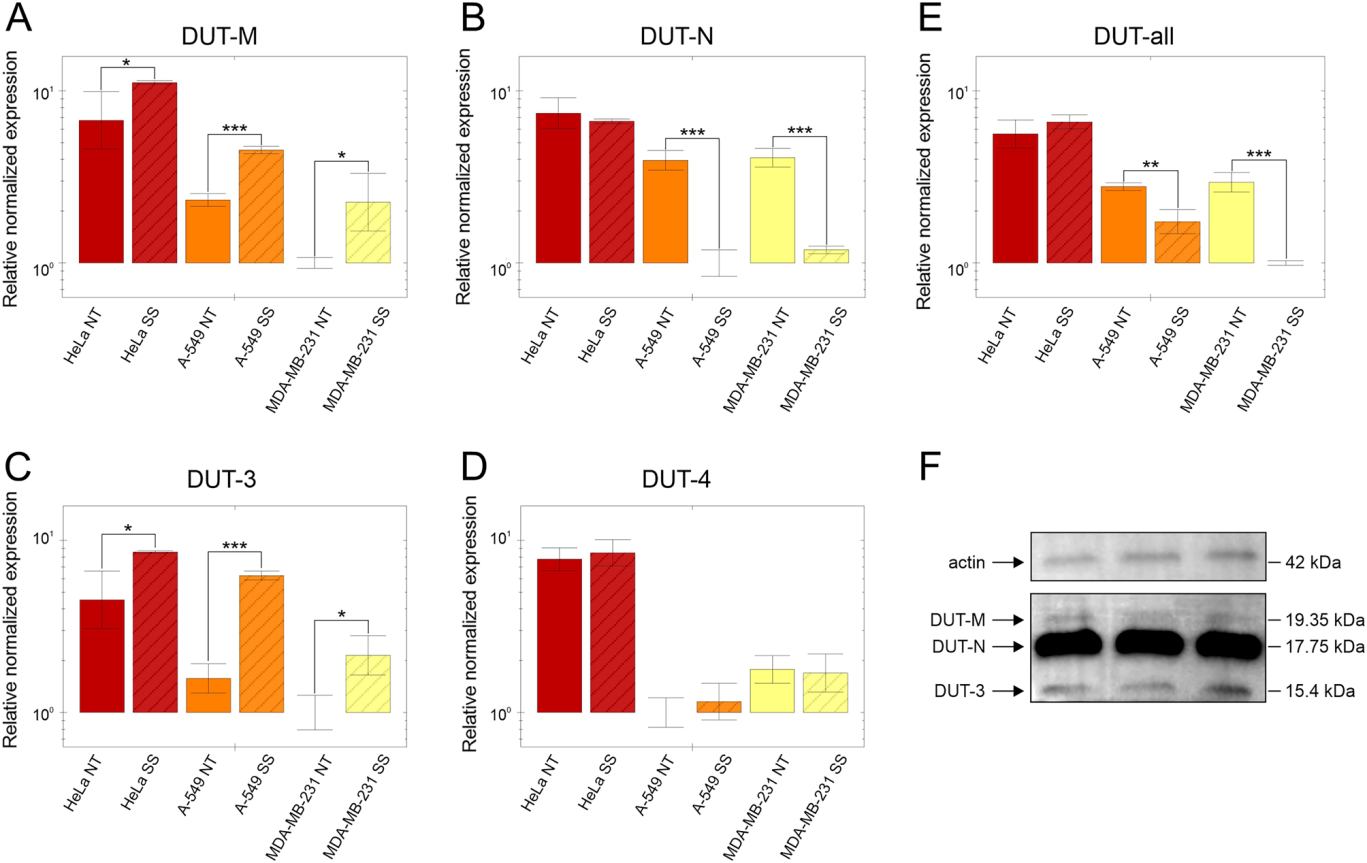
Relative normalized expression of the dUTPase isoforms (**A**) DUT-M, (**B**) DUT-N, (**C**) DUT-3, (**D**) DUT-4 and (**E**) the DUT-all target in HeLa (red), A-549 (orange) and MDA-MB-231 (yellow) cell lines upon serum starvation and (**F**) Western blot analysis of the dUTPase proteins in U-937 cell line. (**A-E**) NT, non-treated (plain columns); SS, serum starved (striped columns). The individual colours correspond to the colours used in Table 2. The scale of the y axis is logarithmic with base 10 and is shown uniformly from 1 to 10 in each panel. In each panel the biological group with the lowest expression was set to 1. Error bars show standard deviation of three biological replicates (n = 3) for each cell line. The number of asterisks indicates increasing possibility that gene expression changes upon serum starvation. * *p *< 0.1, ** *p *< 0.01, *** *p *< 0.001 as calculated by the CFX Maestro software. (**F**) Three technical replicate samples for the detection of dUTPase proteins using western blot. On the left side the target dUTPase proteins and the actin protein as reference are depicted with arrows. On the right side the corresponding theoretical molecular weight values are indicated. The individual full-size images are available as Supplementary Figure S8. Individual graphs for (**A**) DUT-M, (**B**) DUT-N, (**C**) DUT-3, (**D**) DUT-4 and for (**E**) DUT-all were created with CFX Maestro 2.0 (Bio-Rad). (**F**) The image was created with Image Lab 4.1 software (Bio-Rad). The figure was assembled using CorelDRAW Graphics Suite 2020 (Corel Corporation).

The gene expression of the dUTPase isoforms as well as the DUT-all target was determined with RT-qPCR analysis comparing the serum starved cells with the non-treated cells (Fig. 5). For normalization, three reference genes—CNOT4, PUM1 and PCBP1—were selected based on our previous article investigating reference genes^19^. The relative normalized expression of the DUT-4 isoform remained constant during serum starvation in all three cell lines investigated. In case of the DUT-M and the DUT-3 isoforms—which are transcribed from the same promoter—the relative normalized expression increased significantly in every case. The DUT-N isoform has the highest expression of the dUTPase isoforms based on the amplification curves observed. Upon serum starvation, the relative normalized expression of the DUT-N isoform remained constant in HeLa cells, however, decreased significantly in A-549 and MDA-MB-231 cells. The relative normalized expression of the DUT-all target changed in the same direction as the DUT-N isoform, however, the extent of change was found to be less. This phenomenon may be due to the fact that the expression of the DUT-M and DUT-3 isoforms is increased upon serum starvation and this can compensate for the decrease in the expression of the DUT-N isoform. The relative expression values along with the error bars for the serum starved and non-treated samples and the *p*-values are summarized in Supplementary Table S2.

A major finding from the analysis of the effects of serum starvation on the expression levels of dUTPase isoforms is that starvation-induced perturbations occur in cell-line specific manner. Importantly, as shown in Supplementary Figure S7, in all cell lines we have observed the starvation-induced cell cycle arrest in similar extents. Hence, the observed different perturbations in the expression levels of the dUTPase isoforms are not due to differences in cell cycle arrest. The most striking result is that the HeLa cell line shows a strong resilience against serum starvation and keeps the DUT-N isoform at a high expression level, in contrast to the other cell lines (Fig. 5). The main concept described in the literature is that dUTPase has a dual role: it is essential both to remove dUTP for dNTP pool sanitization and to produce dUMP for de novo thymidylate synthesis. In agreement with this, the most abundant isoform DUT-N is expressed mostly during the S phase of the cell cycle and in a growth-dependent manner similarly to other members of the nucleotide precursor biosynthesis and of the DNA replication pathway^7,35–37^. During cell cycle arrest, proliferation stops, thus cells do not require dNTPs for replication. However, repair synthesis can still occur. The observed decrease in the expression of the DUT-N isoform upon serum starvation is in agreement with the role of the enzyme. Our present data, however, also indicate that although this scheme may be typical, in some cancer cell lines the close regulation of the dUTPase expression may be lost as cancer cells lose sensitivity to signalling molecules during continuous proliferation. In case of the HeLa cell line, proliferation stops, however, the high expression level of the DUT-N isoform is preserved.

The DUT-M isoform was previously shown to be expressed in a constitutive manner at both the mRNA and protein levels in 34Lu cells^7^. In contrast, we found that in the three cell lines investigated the expression of the DUT-M isoform significantly increased upon serum starvation. This effect may be important for mitochondrial thymidylate biosynthesis and for better preservation of mitochondrial DNA integrity upon serum starvation. We conclude that in that the expression of DUT-N and DUT-M isoforms are regulated by entirely different mechanisms and also depend on the cell lines. We also found that the expression level of the DUT-3 isoform demonstrates changes parallel to the DUT-M expression levels in all cell lines investigated, further arguing for their common promoter. The lack of significant changes in the expression level of the DUT-4 isoform (containing the same NLS as DUT-N) indicates that all the three cell lines we have investigated in this respect presumably preserve nuclear localization of dUTPase even in starvation.

### Western blot analysis of the dUTPase isoforms

Besides determining the mRNA expression of dUTPase isoforms, we also investigated the protein expression of the isoforms using western blot analysis (Fig. 5F) using 16% acrylamide gel. The original image with 1 s exposure time, the image with 40 s exposure time, and the merged image with the protein ladder are available as Supplementary Figure S8. We used the cell line U-937, which has the highest expression of the DUT-N and DUT-4 isoforms while also having high expression of the DUT-M and DUT-3 isoforms compared to other cell lines investigated in this study. We used actin as reference. We demonstrated three distinct bands corresponding to the DUT-M, the DUT-N and the DUT-3 isoforms—in order of decreasing molecular mass. We calculated the theoretical molecular weight of the dUTPase isoforms using the Protein Molecular Mass tool (https://www.bioinformatics.org/sms/prot_mw.html), moreover, we also calculated the empirical values for the three detected isoforms based on the bands of the protein ladder. The DUT-N isoform has the highest mRNA expression and the highest protein expression as seen as an immense band on the blot. The theoretical molecular mass of the DUT-4 isoform (17.83 kDa) is almost equal to the theoretical molecular mass of the DUT-N isoform (17.75 kDa), therefore, the DUT-4 isoform cannot be detected using western blot technique. The theoretical and the empirical molecular mass of the DUT-M isoform (19.35 kDa and 19.38 kDa, respectively) and of the DUT-N isoform (17.75 kDa and 17.68 kDa, respectively) are reasonably close. The empirical molecular mass (15.41 kDa) of the third detected band is essentially the same as the theoretical molecular mass (15.4 kDa) of the DUT-3 isoform confirming the identity of this novel isoform.

## Conclusion

**Figure 6:**
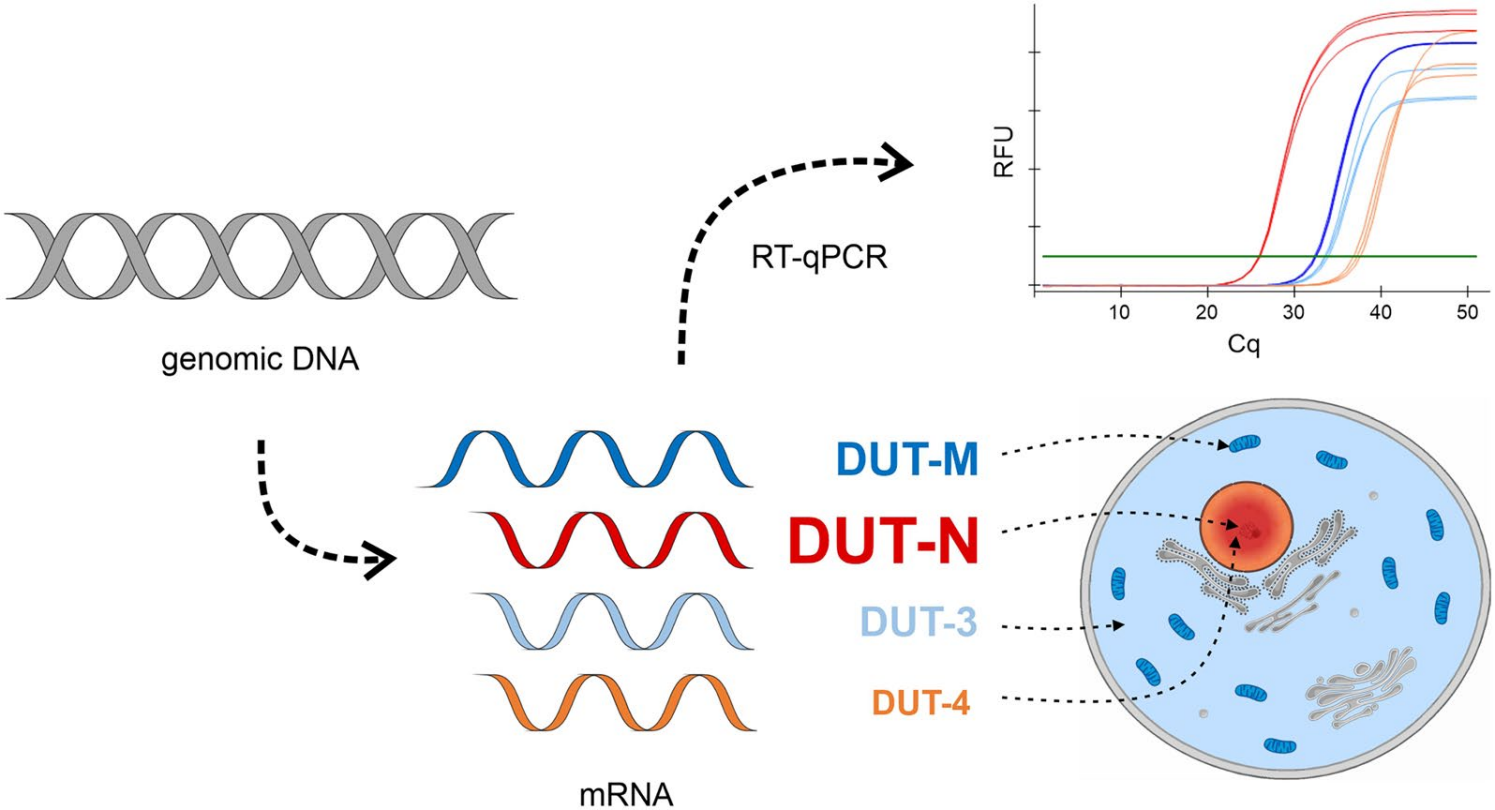
Schematic figure illustrating the main aspects of this study. Dark blue colour indicates the DUT-M isoform, red colour indicates the DUT-N isoform, and the novel isoforms DUT-3 and DUT-4 are coloured light blue and orange, respectively. In the upper right corner representative amplification curves are shown. The font size of the name of the isoforms corresponds to their relative expression levels. The hypothetical cellular localisation of the dUTPase isoforms is depicted by dashed lines. The figure was created using CorelDRAW Graphics Suite 2020 (Corel Corporation).

dUTPase is a ubiquitous enzyme present in all eukaryotic organisms investigated so far from plants through yeasts to animals^34^. The essentiality of this enzyme has been shown through the use of knock-out models while knock-down models demonstrate auxotrophy or sensitivity to DNA damaging agents^4,35–47^. In contrast to its indispensable nature, the isoform distribution of dUTPase was investigated in only a few model organisms. In mouse, one nuclear and one mitochondrial isoform of dUTPase were described—similar to humans^6,7,27^. In *Drosophila melanogaster,* one nuclear and one cytoplasmic dUTPase isoform was shown to exist^48,49^. In *Saccharomyces cerevisiae,* only one bifunctional dITP/dUTP diphosphatase was shown to be present, however, its localization was not studied^50^. In *Dictyostelium discoideum,* only one isoform of dUTPase was identified which localizes solely to the mitochondria^51^. In this work we have identified two additional isoforms for the enzyme dUTPase at the mRNA level in a wide variety of human cell lines of different origins (Fig. 6). Importantly, we identified one of these novel isoforms at the protein level, which lacks any organellar localization signal (DUT-3). Our data now suggest that the presence of dUTPase in the cytoplasm may be a more general phenomenon and not just an exceptional case in the fruit fly. Actually, since dNTPs can freely diffuse through the nuclear pore, there is no explicit and straightforward need for the dUTPase enzyme to be nuclear. However, nuclear presence of this sanitizing enzyme may provide more efficient control of dUTP elimination during DNA synthesis and dUTPase may also interact with other nuclear proteins^52^.

In light of our present work, the dUTPase repertoire in human cells is more extended than previously thought and it includes four isoforms under the regulation of three different promoters according to our results. It remains to be elucidated whether a further variety of dUTPase isoforms can be identified in other species, as well. One promoter reacts sensitively to starvation stress thereby reducing the nuclear and the total dUTPase mRNA levels up to fivefold. However, in the HeLa cell line this strong regulation is lost, potentially enabling better control against DNA uracilation even in resting state (e.g. in repair synthesis). Among the three promoters the one driving the synthesis of DUT-4 nuclear dUTPase isoform shows the most constitutive character, strengthening the importance of the nuclear presence of dUTPase.

## Materials and methods

### Cell lines used in this study

Our aim was to use popular cell lines widely used in numerous studies in the literature. Previously, we summarized the aspects used for the selection of 13 cancer and 7 normal human cell lines^19^, the exact same cell lines were used in this study, as well. The cancer cell lines include HeLa, MCF-7, A-549, K-562, HL-60(TB), HT-29, MDA-MB-231, HCT 116, U-937, SH-SY5Y, U-251MG, MOLT-4 and RPMI-8226, while the normal cell lines include HEK293, MRC-5, HUVEC/TERT2, HMEC, HFF-1, HUES-9 and XCL-1.

### Cell culture

Cell lines HEK293 (CRL-1573), HeLa (CCL-2), SH-SY5Y (CRL2266), U-937 (CRL-1593.2) and the human foreskin fibroblast cell line HFF-1 (SCRC-1041) were purchased from ATCC. Cell lines A-549, HCT 116, HL-60(TB), HT-29, K-562, MCF-7, MDA-MB-231, MOLT-4, MRC-5, and RPMI-8226 were obtained from the National Cancer Institute’s Developmental Therapeutics Program (National Institutes of Health). HMEC cells immortalized with TERT were purchased from the Francis Crick Institute Cell Services Department. HUVEC/TERT2 and MRC-5 were a generous gift from József Tóvári. The human pluripotent stem cell line HUES-9 was kindly provided by Douglas Melton (HHMI). The induced pluripotent stem cell line XCL-1 reprogrammed from CD34 + cord blood cells by episomal vectors, were obtained from XCellScience (Novato, CAXIP-001-1 V). A-549, HCT 116, HEK293, HeLa, HL-60(TB), HT-29, K-562, MCF-7, MDA-MB-231, MOLT-4, RPMI-8226, SH-SY5Y, U-251MG and U-937 cells were cultured in Roswell Park Memorial Institute (RPMI) 1640 medium (Gibco, 72400-021) supplemented with 10% heat-inactivated fetal bovine serum (FBS) (Gibco, 10500064) and 1% Penicillin Streptomycin (Gibco, 15140-122). HFF-1 cells were maintained on gelatine (Sigma) coated plates in DMEM-glutamax medium completed with 10% FBS (Thermo Scientific). HMEC cells were cultured in MEGM Mammary Epithelial Cell Growth Medium BulletKit (Lonza, CC-3150). HUVEC/TERT2 cells was cultured in EBM-2 Endothelial Cell Growth Basal Medium-2 (Lonza, 00190860) supplemented with components from the EGM-2 Endothelial SingleQuots Kit (Lonza, CC-4176). MRC-5 cells were cultured in Dulbecco’s Modified Eagle Medium (DMEM) (Gibco, 11995-065) supplemented with 20% FBS and 1% Penicillin Streptomycin. HUES-9 and XCL-1 cells were maintained on Matrigel (Corning) coated six well plates in mTeSR medium (Stemcell Technologies). All cell lines were cultivated at 37 °C in a humidified incubator with 5% CO2 atmosphere. All cell cultures were free of mycoplasma as determined by PCR. Adhesion cell lines were passaged when the culture reached 40–50% confluency to avoid contact inhibition. Suspension cell lines were passaged every 2–3 days. For RNA extraction, cells were collected after 2 days of passage.

For serum starvation treatment, cells were cultured in RPMI 1640 medium (Gibco, 72400-021) supplemented with 1% Penicillin Streptomycin (Gibco, 15140-122) and without 10% heat-inactivated FBS. Cells after serum starvation were washed with Phosphate buffered saline solution (PBS) (Sigma, P3813), trypsinized and resuspended in fresh medium supplemented with 3 mM EDTA (Sigma, E9884) —and in case of serum starved cultures—10 mg/ml bovine serum albumin (BSA, Sigma, A7906). EDTA and BSA were dissolved in MilliQ water and the stock solutions were sterile filtered with Millex-GP Millipore Express PES Membrane Filter Unit (Millipore). Cells were centrifuged at 200 g for 5 min using Eppendorf MiniSpin centrifuge (type 5452) and washed with PBS.

### RNA extraction and quality control

Adhesive cells were trypsinized with Trypsin–EDTA solution (Sigma, T3924) and resuspended in fresh medium. Suspension cell cultures and trypsinized adhesive cells were centrifuged at 200 g for 5 min using an Eppendorf MiniSpin centrifuge (type 5452) and washed twice with PBS. The pellet was resuspended in RLT buffer (a part of the Qiagen RNeasy Plus Mini kit) supplemented with 1% beta-Mercaptoethanol (Merck) and lysed by vortexing for 1 min using sterile glass beads. The lysed samples were kept at − 20 °C until further processing. RNA was extracted with Qiagen RNeasy Plus Mini kit following the manufacturer’s recommendations. On-column DNase digestion was performed with RNase-Free DNase Set (Qiagen, 79254). RNA was eluted in 50 µl nuclease-free water (Ambion). The concentration and the purity as indicated by the 260/280 and 260/230 ratios were determined with NanoDrop ND-2000. To ensure that equal RNA quantity is measured in the following reverse transcription reaction, the concentration of all RNA samples were set to 24 ng/µl and verified with NanoDrop. Agarose gel electrophoresis was performed in order to assess the integrity of the RNA samples and potential genomic DNA contamination using 1% agarose (Sigma, A9539) and TBE running buffer. Equally 600 ng RNA was mixed with gel loading dye (New England Biolabs, B7024S) and loaded into the wells of the gel. GeneRuler 1 kb Plus DNA Ladder (Thermo Scientific, SM1331) was used as marker. Gel Doc XR + Imager (Bio-Rad) was used for imaging. RNA samples were kept at − 80 °C until further processing.

### Reverse transcription

To assess the suitability of the RT reaction, the Maxima First Strand cDNA Synthesis Kit for RT-qPCR (Thermo Scientific, K1642) and the High-Capacity cDNA Reverse Transcription Kit (Applied Biosystems, 4,368,814) were compared. The kits were used according to the manufacturer’s recommendations and for the Maxima First Strand cDNA Synthesis Kit for RT-qPCR, the RT reaction was performed at 65 °C for 30 min. A series of 6 point twofold dilutions was prepared from the RNA extracted from HCT 116 cells and introduced to the RT reaction with the starting concentration of 1600 ng/µl. For further experiments, the Maxima First Strand cDNA Synthesis Kit for RT-qPCR was used with 200 ng RNA introduced to the reaction. The RT reaction was performed in Applied Biosystems GeneAmp PCR system 2700. The cDNA samples were kept at − 20 °C until further processing.

### Primer design

The transcription start site of the DUT-4 isoform lies upstream from that of the other isoforms (Fig. 1A). For the detection of the DUT-4 isoform an intron-flanking forward primer was designed in its first exon shown in orange. The DUT-M isoform probably shares the same promoter with the DUT-3 isoform as the transcription of these isoforms starts in the same position coloured in light blue. The transcription of the DUT-M isoform continues with the sequence coloured in dark blue, while this sequence which corresponds to the mitochondrial targeting sequence is absent in the DUT-3 isoform. The intron-flanking forward primer for the DUT-M isoform was designed in this exon. The transcription of both the DUT-M and the DUT-3 isoform continues with the sequence coloured in green. The intron-spanning forward primer for the DUT-3 isoform is coloured in grey. The transcription of the DUT-N isoform starts with the sequence coloured in red and continues with the common sequence. For the detection of the DUT-N isoform an exonic primer design was applied. Figure 1B shows the protein sequence of the dUTPase isoforms. All isoforms contain the core nuclear localisation signal (KRAR)^10^ except the DUT-3 isoform, whose translation initiation site lies downstream from the bold green common sequence.

We also aimed to determine the overall dUTPase mRNA expression level (DUT-all). As the common coding region of all dUTPase isoforms is highly similar to the *Homo sapiens* zinc finger protein 534 (ZNF534) transcript variant 3 (NCBI RefSeq ID: NM_001291368.4) and 4 (NCBI RefSeq ID: NM_001291369.4), the primer pairs for the common sequence were designed to be located at the 3’ UTR region. For the specific amplification of the DUT-3 isoform, the forward primers were designed to be located on exon-exon junction (intron spanning design). In case of DUT-4 and the DUT-M isoforms, the forward and reverse primers were separated with an intron (intron-flanking design). The primer design of the aforementioned isoforms excluded the possibility of amplifying genomic DNA contamination. The DUT-N isoform of dUTPase was determined using an exonic primer design. For all targets three or four primer pairs were designed and compared with temperature gradient PCR coupled with melting curve analysis and agarose gel electrophoresis to ensure the specificity of the PCR products. Agarose gel electrophoresis was performed during the optimisation, while melting curve analysis was carried out routinely after each PCR reaction. Specific products were indicated as a single sharp band on agarose gel and characterized by a single peak with melting curve analysis. In case more than one primer pair generated specific products, the one with the lowest Cq value was selected for further experiments.

To design primer pairs the NCBI primer designing tool was used^53^. PCR product length was limited to 120 base pairs (bp)^54^. The melting temperatures of the primers were set to be in the range of 60–63 °C. Specificity was investigated with BLAST with the following parameters: at least 5 total mismatches to unintended targets, including at least 3 mismatches within the last 5 bps at the 3' end. Targets with more than 6 mismatches were ignored for the specificity check. The primers were ordered from Merck with desalting purification in a dry format and dissolved in nuclease-free water following the recommendation to make 100 µM solutions. The concentration of the primer solutions were checked with NanoDrop.

### Quantitative polymerase chain reaction (qPCR)

The qPCR reaction was performed in 10 µL final volume using MyTaq HS Mix (Bioline, BIO-25046), Evagreen dye (Biotium, 31000), nuclease-free water, cDNA template, and appropriate primers. In each qPCR reaction 0.1 µl cDNA sample was used and the final concentrations of all primers were 500 nM. Three technical replicates were used for every sample and every target gene. Two technical replicates of no template control (NTC) reaction were measured for each target on each plate. No reverse transcription control (NRT) was measured randomly for 25% of the samples. NRT controls were prepared from the RNA samples using nuclease-free water instead of the RT enzyme and the reaction buffer. The difference between the Cq values of the NRT/NTC and the samples were higher than 10 in most cases, and higher than 5 in all cases.

Clear Hard-Shell 96-Well PCR Plates (Bio-Rad) and Microseal 'B' PCR Plate Sealing Film (Bio-Rad) were used. Thermal cycling and detection was performed in CFX96 real-time PCR detection system (Bio-Rad). Thermal cycling protocol includes initial denaturation and hot-start polymerase activation at 95 °C for 5 min followed by 50 cycles of denaturation at 95 °C for 30 s and annealing/extension at 63 °C for 30 s. After amplification, melting curve analysis was performed from 60 °C to 95 °C with an increment of 0.5 °C every 5 s. For comparing the melting curves of the PCR products used in the identification of the dUTPase isoforms, the temperature increment was set to 0.2 °C.

### Determination of PCR efficiency

For the determination of PCR efficiency values using PCR products, RNA samples extracted from three biological replicates of HCT 116 cells were pooled and introduced in reverse transcription reactions for each target followed by amplification with qPCR as described above. The PCR products were analysed with agarose gel electrophoresis using 2% agarose and TAE running buffer. The PCR products were purified from the gel using NucleoSpin Gel and PCR Clean-up (Macherey–Nagel, 740609) according the manufacturer’s recommendation. The concentrations of the solutions were determined with NanoDrop. Series of 7 point tenfold dilutions were prepared and each concentration point was introduced in qPCR reactions in the range of final concentration from 100 fg/µl to 0.0001 fg/µl with three technical replicates. The Cq values were plotted against the logarithm with base 10 of the concentration and the slope of the curves and regression coefficients were determined and the PCR efficiency values were calculated with the formula E(%) = [10^(1/-slope)-1]*100%. The PCR efficiency values obtained from the measurements with PCR products were used for further calculations.

We also determined the PCR efficiency values using cDNA samples for two dUTPase targets, the DUT-N and DUT-all and—in our previous article—for the reference gene targets IPO8, PUM1, SNW1. For this purpose cDNA derived from biological replicates of HCT 116 cells were pooled and 6 point fourfold dilution series was prepared. The most concentrated points contained 0.3 µl cDNA in each well. Three technical replicates were applied for each target and each concentration point. The results were evaluated as discussed above.

### Assessment of the specificity of PCR

To verify the specificity of the PCR products initially, cDNA derived from biological replicates of HCT 116 cells were pooled and introduced in qPCR reactions. Sanger sequencing was performed for longer PCR products for each dUTPase isoforms. The following reverse primer—along with the appropriate forward primers for each dUTPase target—was used to generate the longer PCR products: 5’-TGGTATTGTGTAATCATAGGCACTGT-3’. Using these targets as templates a second round nested PCR was performed and the PCR products were compared to the PCR products from single-round PCR reactions with agarose gel electrophoresis and melting curve analysis. For the agarose gel electrophoresis 2% agarose and TAE running buffer were used. For each gene 2–4 µl of PCR products were mixed with loading dye and loaded on the gel. GeneRuler 1 kb Plus DNA Ladder and GeneRuler 100 bp Plus DNA Ladder (Thermo Scientific, SM0321) were used as markers. Gel Doc XR + Imager was used for imaging. Melting curve analysis was performed after every amplification. In case the melting curves showed aspecific product formation, the wells were excluded from the analysis.

### Flow cytometry

Cell cycle phase distribution was analysed with flow cytometry. Cells were treated with 5-Ethynyl-2′-deoxyuridine (EdU) in 10 µM concentration for 20 min. Cells were collected as discussed above. Staining of cells for DNA synthesis was performed using Click-iT™ Plus EdU Alexa Fluor™ 488 Flow Cytometry Assay Kit, (Invitrogen, C10632) according to the manufacturer’s recommendations. Cells were also stained for DNA content with 10 µg/ml Propidium Iodide (Thermo Scientific) and 20 µg/ml RNase A (Sigma, 10109142001 dissolved in 10 mM Tris-HCl, 15 mM NaCl, pH = 7) in 500 µl PBS and incubated at room temperature for 30 min protected from light. Samples were analysed with Attune NxT flow cytometer (Thermo Fischer Scientific Waltham, MA, US). The Click-iT™ Plus EdU Alexa Fluor™ 488 signal was detected with 488 nm excitation and 530/30 nm emission in the BL1 channel. The Propidium-Iodide signal was detected with 488 nm excitation and 695/40 nm emission in the BL3 channel. Analysis of the results was performed using Attune NxT 3.2.1 software.

### Western blot analysis

Cells were lysed by vortexing for 1 min in RIPA buffer (150 mM sodium-chloride, 1% Nonidet P-40 Substitute, 0.5% sodium deoxycholate, 0.1% SDS, 50 mM TRIS HCl, 2 mM DTT, 1 mM PMSF, 5 mM benzamidine, 2 mM EDTA) supplemented with cOmplete ULTRA Tablets (Roche) and PhosSTOP (Roche). The lysate was sonicated twice for 3 min with degas function at 4 °C (Elma S 30 H Elmasonic) and centrifuged for 5 min at 4 °C at 10621 g (Eppendorf Centrifuge 5804 R). The supernatant was collected and stored at − 20 °C until further processing. The protein concentration of the samples were determined with Pierce BCA Protein Assay Kit (Thermo Scientific, 23227) using BioTek Synergy Mx Microplate Reader. 15 µg total protein was loaded in each well after adding 3.5 µl loading buffer 5X (250 mM Tris-HCl, 50% glycerol, 10% DTT, 10% SDS, 0.05% bromophenol blue, pH = 6.8) and RNase-free water in final volume of 17.5 µl. The samples were incubated at 95 °C for 5 min and loaded to 16% polyacrylamide gel. GRS Protein Marker Multicolor (GRiSP, GLP01.0500) was used as protein ladder. Electrophoresis was carried out in the Mini-PROTEAN Electrophoresis System (Bio-Rad) in Tris-Glycine SDS Running Buffer (25 mM Tris–HCl, 20 mM glycine, 0.1% SDS) at 200 V for 90 min. For the transfer Immobilon-P 0.45 µm pore size PVDF transfer membrane (Merck, IPVH09120) was used with transfer buffer (10 mM CAPS, 15% methanol, pH = 11) at 250 mA for 3 h. The membrane was dried on filter paper and cut after the transfer and placed in TBS-T (25 mM TRIS HCl, 140 mM NaCl, 3 mM KCl, 0.05% Tween-20, pH = 7.4). Blocking and immunostaining was performed in 5% skimmed milk powder in TBS-T. After 1 h blocking, incubation with primary antibodies was performed overnight (Anti-Actin (20–33) polyclonal antibody produced in rabbit (Sigma, A5060), Anti-dUTPase monoclonal antibody produced in rat (Sigma, SAB4200044)). After washing the membranes three times for 10 min with TBS-T, incubation with secondary antibodies was performed for 2 h protected from light (anti-rabbit IgG (GE Healthcare, Na934vs) for actin, anti-rat IgG (Sigma, A9542) for dUTPase). After washing the membranes again three times for 10 min with TBS-T, membranes were placed in TBS buffer until imaging. Bands were visualized with Immobilon Western Chemiluminescent HRP Substrate (Merck, WBKLS0100). Imaging was performed with ChemiDoc MP Imaging System (Bio-Rad).

### Data analysis

For the gene expression analysis, the CFX Maestro 2.0 (Bio-Rad) software was used (URL: https://www.bio-rad.com/en-us/product/cfx-maestro-software-for-cfx-real-time-pcr-instruments). The threshold value was set uniformly to 500 relative fluorescence unit (RFU) for each plate measured. Gel images were captured with Image Lab 4.1 software (Bio-Rad) (URL: https://www.bio-rad.com/en-hu/product/image-lab-software). Graphs were created with OriginPro 2018 (OriginLab Corp.) (URL: https://www.originlab.com/2018). CorelDRAW Graphics Suite 2020 (Corel Corporation) was used for creating figures from individual graphs (URL: https://www.coreldraw.com/en/product/coreldraw/).

To assess the relation of the relative normalized expression of the dUTPase isoforms to the overall expression, pairwise comparisons were carried out between each normal cell line and the group of cancer cell lines. The relative normalized expression data were extracted from CFX Maestro 2.0 (Bio-Rad). The logarithm of the ratio of the relative normalized expression of each isoform to the overall expression (measured with the DUT-all target) was calculated and termed as ratio indicator. As the variance of the three biological replicate samples measured in different cell lines is not equal, we performed the non-parametric Kruskal–Wallis analysis followed by Conover-Iman pairwise comparisons with Bonferroni correction using XLSTAT (Lumivero). After Bonferroni correction, *p* values were defined to be significant below 0.0018. For the serum starvation experiment, the *p*-values were calculated by CFX Maestro software.

## Supplementary Information

Supplementary Information.

## Data Availability

No datasets were generated or analysed during the current study. Upon request raw data is available via the email address vertessy.beata@ttk.hu.
