# Supplementary material for: Discovery of two new isoforms of the human DUT gene
Source: Sci Rep. 2023 May 12;13:7760. doi: 10.1038/s41598-023-32970-1 (PMC10181998; doi:10.1038/s41598-023-32970-1)
Supplement: Supplementary file 1 — Supplementary Information. [file 41598_2023_32970_MOESM1_ESM.docx]

**Supplementary Information**

**Discovery of two new isoforms of the human DUT gene**

Gergely Attila Rácz^1,2*^, Nikolett Nagy^2,3^, György Várady^2^, József Tóvári^4^, Ágota Apáti^2^, Beáta G. Vértessy^1,2*^

Affiliations

^1^Department of Applied Biotechnology and Food Sciences, Faculty of Chemical Technology and Biotechnology, Budapest University of Technology and Economics, Műegyetem rkp. 3., H-1111 Budapest, Hungary

^2^Institute of Enzymology, Research Centre for Natural Sciences, Eötvös Loránd Research Network, Budapest, Hungary

^3^Doctoral School of Biology, Institute of Biology, ELTE Eötvös Loránd University, 1117 Budapest Pázmány Péter sétány 1/C, Budapest, Hungary

^4^Department of Experimental Pharmacology, National Institute of Oncology, Ráth Gy. u. 7-9, H-1122, Budapest, Hungary

*Corresponding authors

Correspondence and requests for materials should be addressed to Beáta G. Vértessy (email: vertessy@kutatok.org). Correspondence may also be addressed to Gergely Attila Rácz (racz.gergely@ttk.hu).


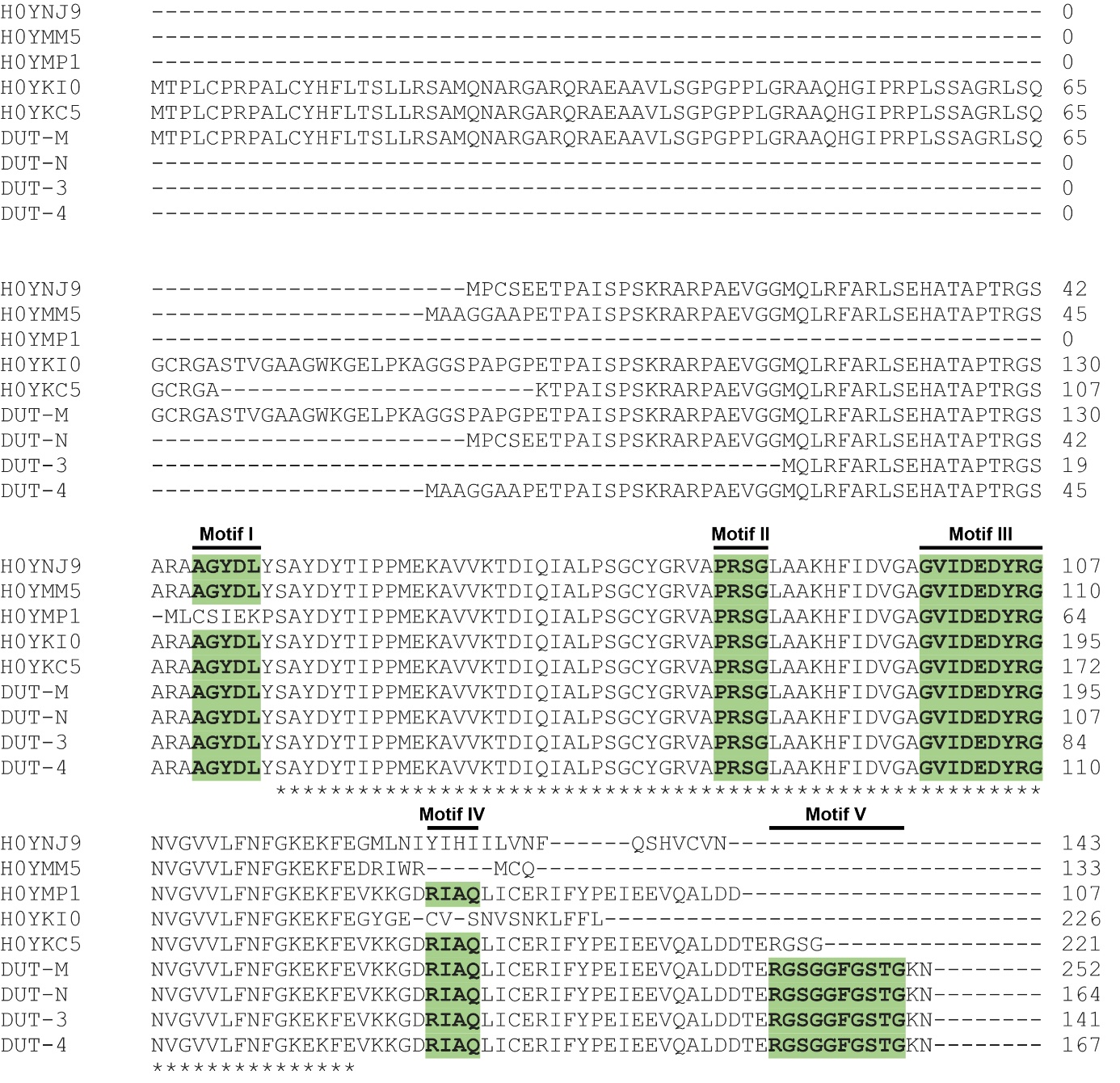


**Supplementary Figure S1.** Multiple protein sequence alignment of the dUTPase isoforms present in the UniProt database made with the Clustal Omega online tool (<https://www.ebi.ac.uk/Tools/msa/clustalo/>). Conserved motifs are shown in bold and highlighted with green colour. The figure was assembled using CorelDRAW Graphics Suite 2020 (Corel Corporation).


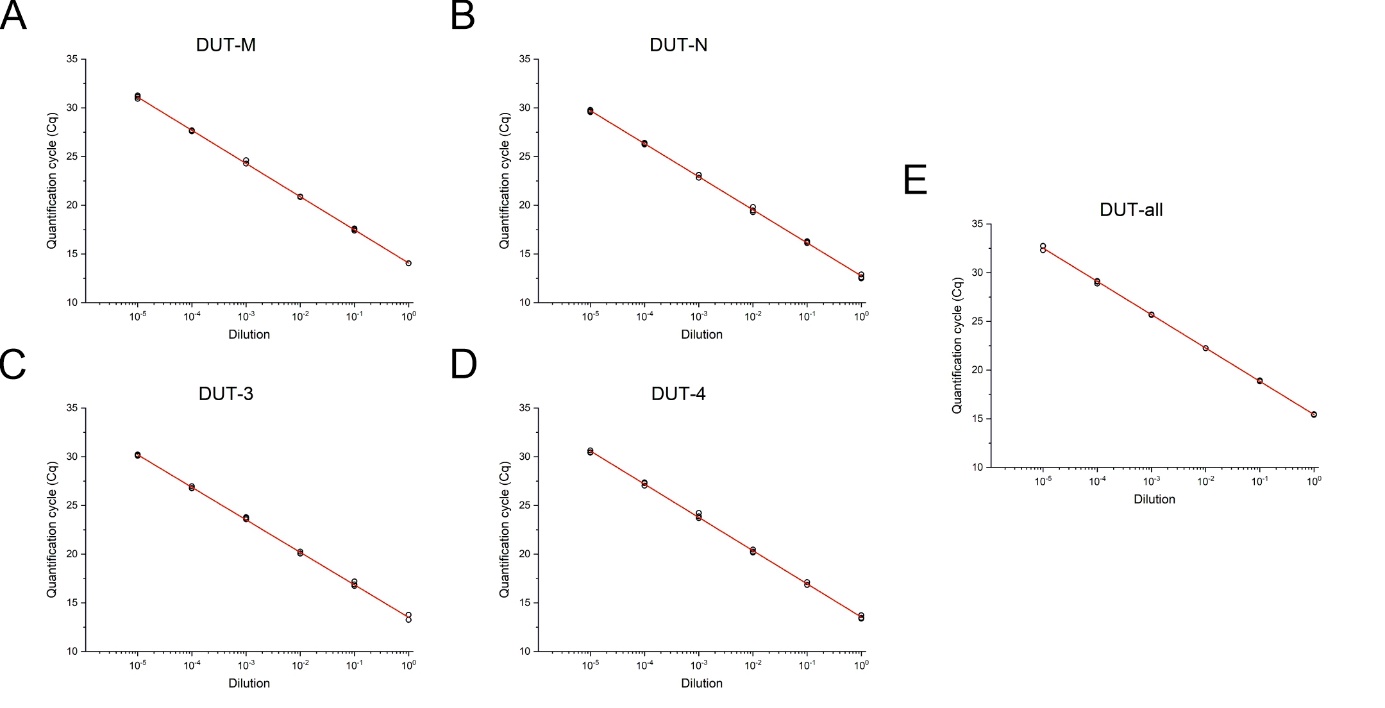


**Supplementary Figure S2.** Dilution curves for determination of PCR efficiency of (**A**) DUT-M, (**B**) DUT-N, (**C**) DUT-3, (**D**) DUT-4 and (**E**) DUT-all. 6 point 10-fold serial dilutions were prepared from PCR products and submitted to qPCR analysis. The Cq values for three technical replicates for each concentration point are marked as hollow circles. Least squares linear regression was performed to the average of the technical replicates in the indicated dilution range. Individual graphs were created with OriginPro 2018 (OriginLab Corp.) and the figure was assembled using CorelDRAW Graphics Suite 2020 (Corel Corporation).


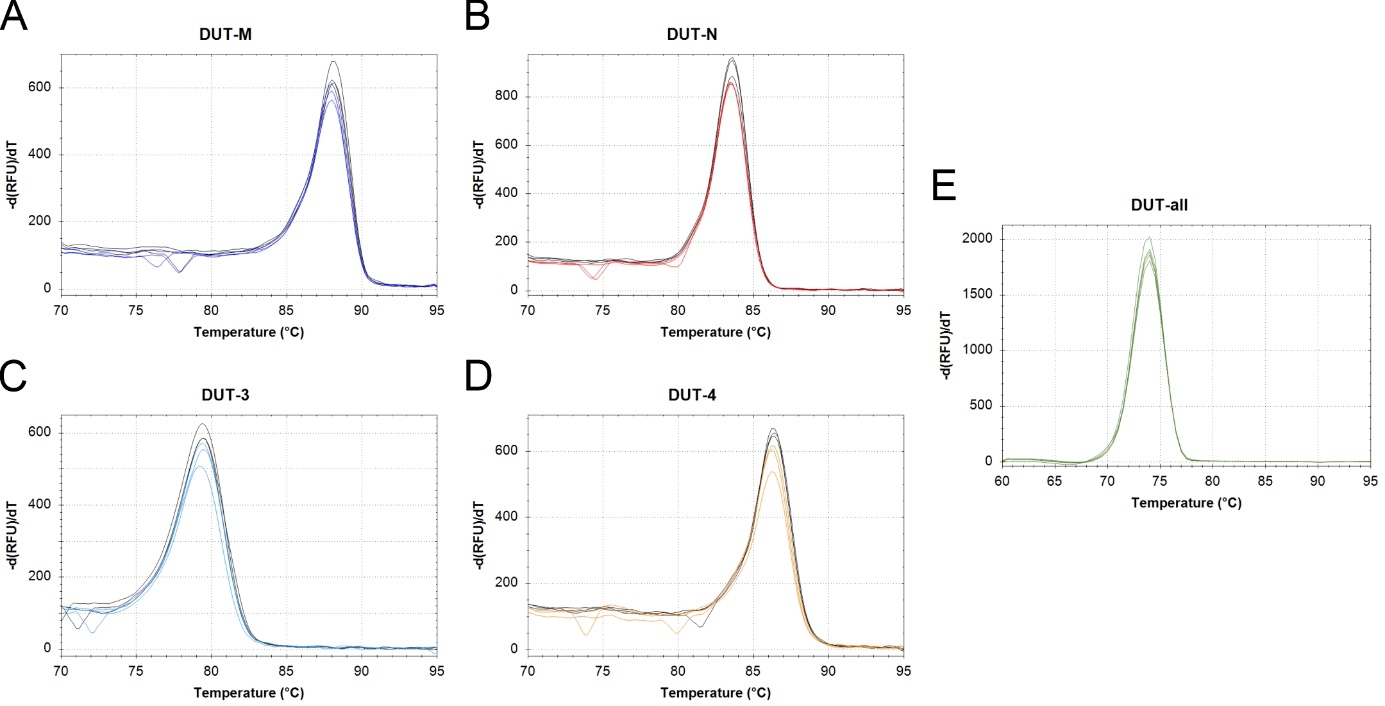


**Supplementary Figure S3.** Melting curve analysis of PCR products of (**A**) DUT-M, (**B**) DUT-N, (**C**) DUT-3, (**D**) DUT-4 and (**E**) DUT-all. Melting curves were detected with a temperature increment of 0.2 °C every 5 seconds. Melting curves derived from single-round PCR are coloured in case of (**A**) DUT-M dark blue, (**B**) DUT-N red, (**C**) DUT-3 light blue, (**D**) DUT-4 orange and (**E**) DUT-all green, while melting curves derived from nested PCR are coloured black. In case of the DUT-all target, melting curves were detected only from single-round PCR. Individual graphs were created with CFX Maestro 2.0 (Bio-Rad) and the figure was assembled using CorelDRAW Graphics Suite 2020 (Corel Corporation).


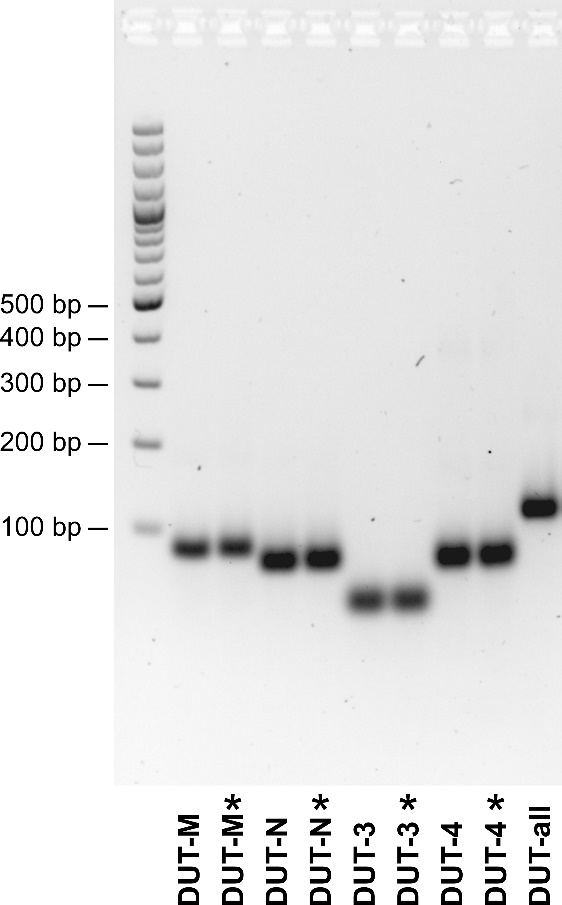


**Supplementary Figure S4.** PCR product quality control with agarose gel electrophoresis of PCR products derived from single-round and nested PCR. Asterisks (*) indicate PCR products from nested PCR. On the left side, GeneRuler 100 bp Plus DNA ladder was used as molecular-weight size marker. The image was created with Image Lab 4.1 software (Bio-Rad) and the figure was assembled using CorelDRAW Graphics Suite 2020 (Corel Corporation).


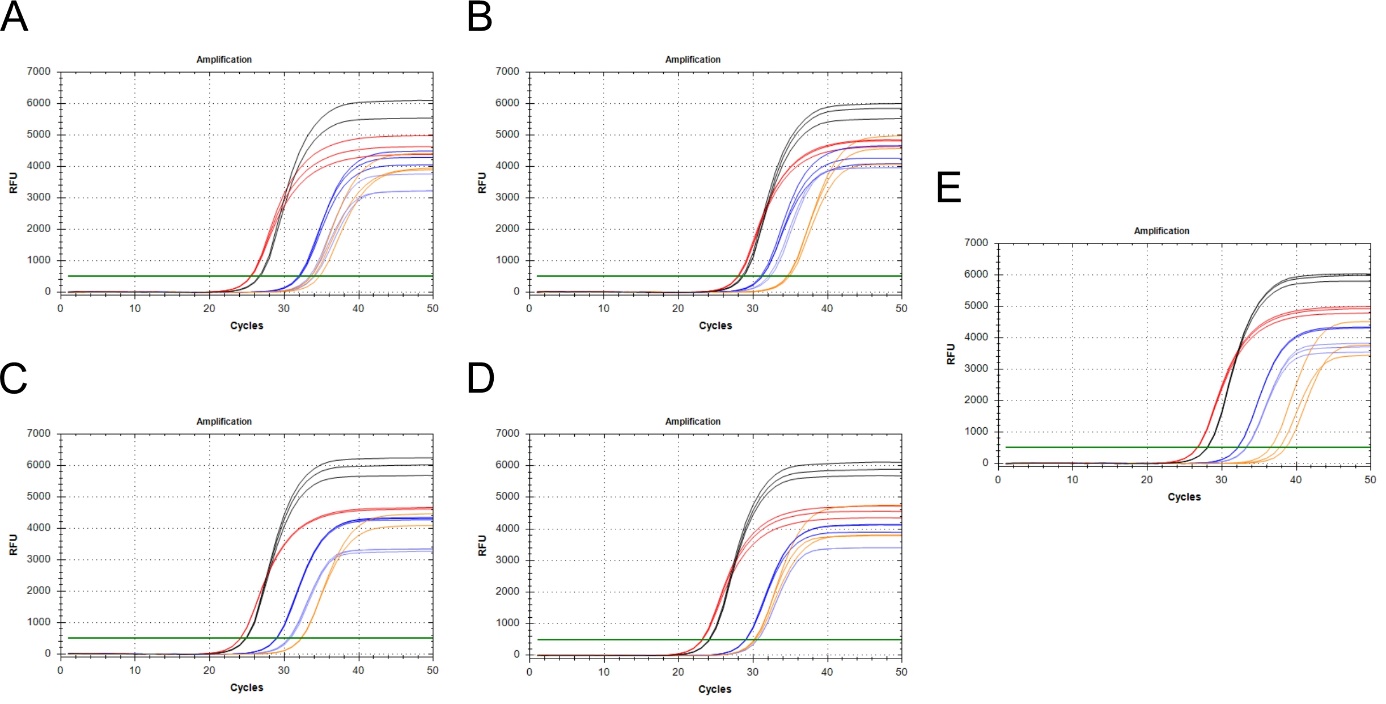


**Supplementary Figure S5.** Amplification curves measuring the expression of the dUTPase isoforms and the DUT-all target. Dark blue colour indicates the DUT-M isoform, red colour indicates the DUT-N isoform, the novel isoforms DUT-3 and DUT-4 are coloured light blue and orange, respectively, and the DUT-all target is shown in black curves. Amplification curves (**A**) for MDA-MB-231 cell line having the lowest expression of the DUT-M and DUT-3 isoforms, (**B**) MRC-5 cell line having the lowest expression of the DUT-N isoform and the DUT-all target, (**C**) U-251MG cell line having the highest expression of the DUT-M and DUT-3 isoforms, (**D**) U-937 having the highest expression of the DUT-N and DUT-4 isoforms and the DUT-all target, (**E**) HCT-116 cell line having the lowest expression of the DUT-N isoform. The image was created with CFX Maestro 2.0 (Bio-Rad) and the figure was assembled using CorelDRAW Graphics Suite 2020 (Corel Corporation).


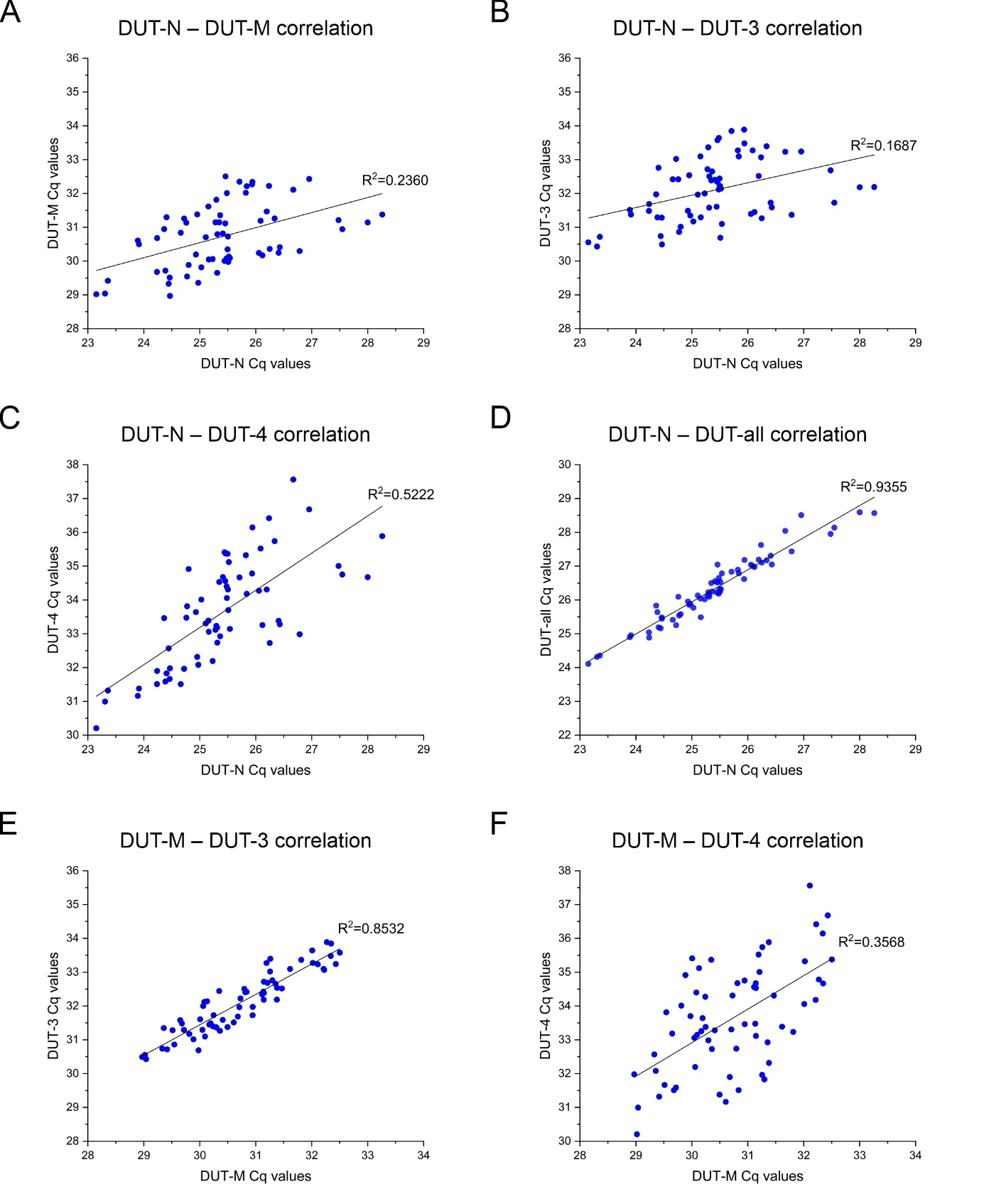


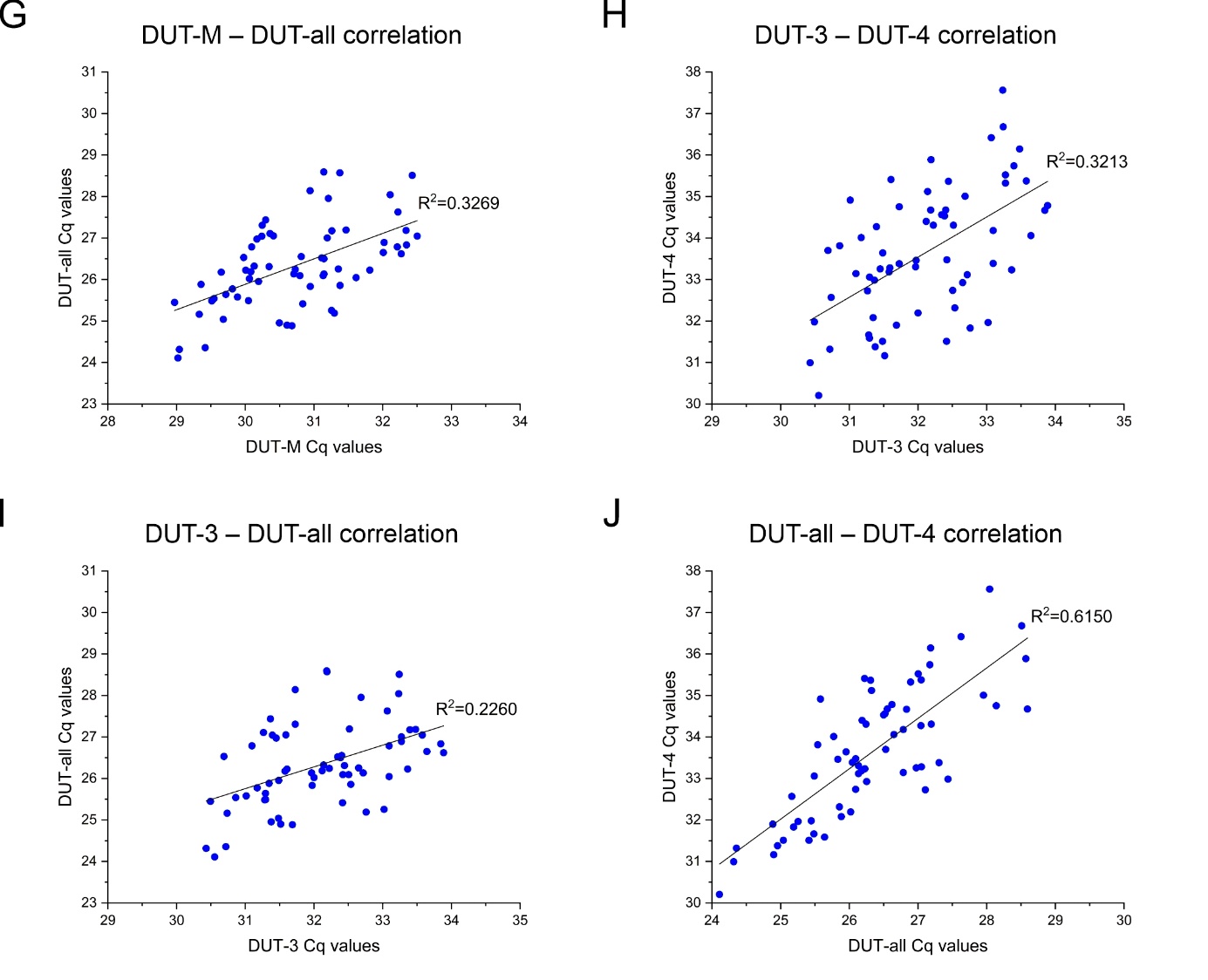


**Supplementary Figure S6.** Correlation analysis of the Cq values of every combination of two targets of the dUTPase isoforms and the DUT-all target (**A-J**). Regression coefficient values were determined by performing least squares linear regression to all data points. The range displayed on the axes is constant in all graphs for comparability. Individual graphs were created with OriginPro 2018 (OriginLab Corp.) and the figure was assembled using CorelDRAW Graphics Suite 2020 (Corel Corporation).


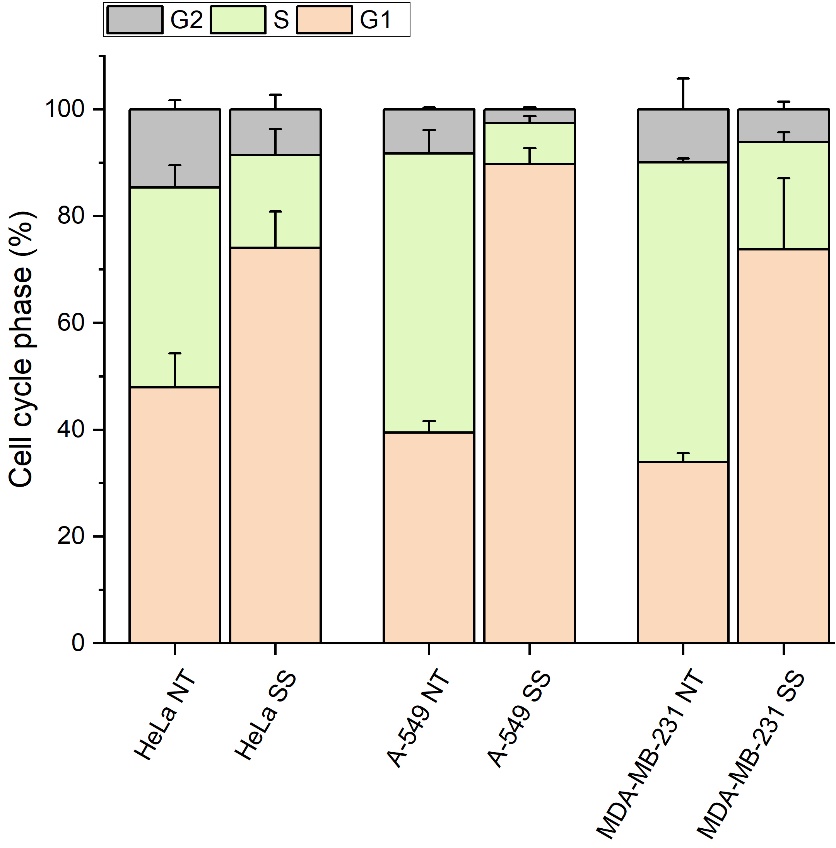


**Supplementary Figure S7.** Cell cycle phase distribution of Hela, A-549 and MDA-MB-231 cell cultures using three biological replicates. Non-treated (NT) and serum starved (SS) cultures were analysed with flow cytometry. The G2 phase is coloured grey, S phase is coloured light green and G1 phase is coloured light orange. Only the upper error bar is depicted. This graph was created with OriginPro 2018 (OriginLab Corp.).


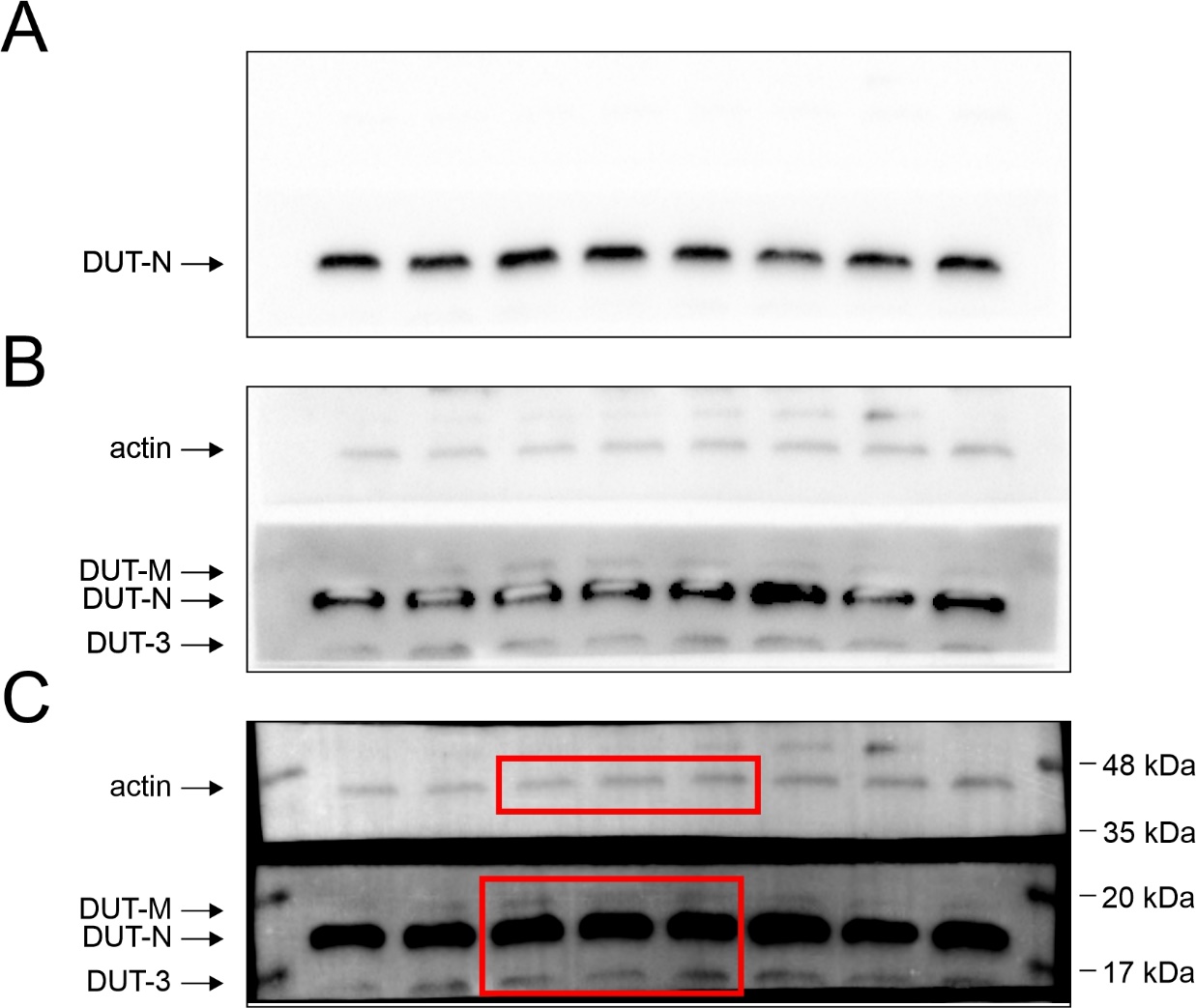


**Supplementary Figure S8.** Western blot analysis of technical replicate samples from U-937 cell line. The membranes were cut before hybridisation with antibodies for actin and dUTPase. (**A**) 1 sec exposure time to visualize the DUT-N isoform. (**B**) 40 sec exposure time to visualize the actin and the dUTPase isoforms. (**C**) Merged image of the 40 sec exposure time image and of the protein ladder detected with colorimetric setting. The red rectangles show the corresponding area depicted in Figure 5F. (**A-C**) On the left side the target dUTPase proteins and actin as reference are depicted with arrows. On the right side the protein ladder bands with the corresponding molecular weight values are indicated. The image was created with Image Lab 4.1 software (Bio-Rad) and the figure was assembled using CorelDRAW Graphics Suite 2020 (Corel Corporation).

**Supplementary Table S1.** Relative normalized expression values and the range of error based on the standard deviation for the dUTPase isoforms and the DUT-all target as calculated by the CFX Maestro software on a base-2 logarithmic scale.

| **Cell line** | **Relative normalized expression (lg) ± Standard deviation (lg)** | | | | |
| --- | --- | --- | --- | --- | --- |
|  | **DUT-M** | **DUT-N** | **DUT-3** | **DUT-4** | **DUT-all** |
| A-549 | 1.204 ± 0.125 | 1.641 ± 0.238 | 0.673 ± 0.335 | 0.885 ± 0.327 | 1.182 ± 0.113 |
| HCT-116 | 0.143 ± 0.400 | 1.702 ± 0.392 | 0.405 ± 0.382 | 0.000 ± 0.877 | 0.817 ± 0.334 |
| HEK | 1.828 ± 0.119 | 1.823 ± 0.108 | 1.200 ± 0.160 | 0.967 ± 0.372 | 1.548 ± 0.053 |
| HeLa | 2.623 ± 0.488 | 2.449 ± 0.238 | 2.099 ± 0.493 | 3.687 ± 0.266 | 2.096 ± 0.207 |
| HFF | 1.946 ± 0.123 | 1.136 ± 0.330 | 2.305 ± 0.257 | 3.164 ± 0.368 | 0.927 ± 0.237 |
| HL-60 | 1.906 ± 0.186 | 3.748 ± 0.073 | 2.382 ± 0.125 | 4.831 ± 0.156 | 3.352 ± 0.280 |
| HMEC | 2.239 ± 0.232 | 1.296 ± 0.449 | 2.168 ± 0.349 | 2.756 ± 0.447 | 1.064 ± 0.359 |
| HT-29 | 1.095 ± 0.366 | 1.940 ± 0.103 | 1.174 ± 0.222 | 1.506 ± 0.271 | 1.459 ± 0.289 |
| HUES9 | 0.646 ± 0.390 | 1.847 ± 0.233 | 0.568 ± 0.252 | 2.862 ± 0.477 | 1.503 ± 0.217 |
| HUVEC | 2.082 ± 0.482 | 1.560 ± 0.165 | 2.398 ± 0.292 | 2.326 ± 0.184 | 1.071 ± 0.236 |
| K-562 | 0.952 ± 0.351 | 2.406 ± 0.172 | 1.162 ± 0.322 | 2.321 ± 0.269 | 1.644 ± 0.071 |
| MCF-7 | 0.354 ± 0.441 | 1.661 ± 0.285 | 0.472 ± 0.672 | 1.399 ± 0.417 | 1.104 ± 0.232 |
| MDA-MB-231 | 0.000 ± 0.059 | 1.674 ± 0.111 | 0.000 ± 0.378 | 1.676 ± 0.238 | 1.246 ± 0.190 |
| MOLT-4 | 1.319 ± 0.222 | 3.119 ± 0.167 | 1.119 ± 0.160 | 4.490 ± 0.138 | 2.926 ± 0.256 |
| MRC-5 | 1.447 ± 0.060 | 0.000 ± 0.184 | 1.962 ± 0.120 | 1.392 ± 0.551 | 0.000 ± 0.117 |
| RPMI-8226 | 3.027 ± 0.141 | 3.560 ± 0.075 | 2.653 ± 0.155 | 4.884 ± 0.050 | 2.999 ± 0.265 |
| SH-SY5Y | 2.121 ± 0.151 | 2.232 ± 0.125 | 2.199 ± 0.091 | 1.140 ± 0.502 | 1.959 ± 0.122 |
| U-251 | 3.391 ± 0.162 | 3.222 ± 0.168 | 3.282 ± 0.283 | 3.901 ± 0.071 | 3.046 ± 0.395 |
| U-937 | 2.873 ± 0.148 | 4.041 ± 0.061 | 2.901 ± 0.185 | 5.020 ± 0.392 | 3.556 ± 0.040 |
| XCL1 | 0.363 ± 0.340 | 1.950 ± 0.057 | 0.311 ± 0.473 | 2.608 ± 0.375 | 1.551 ± 0.075 |

**Supplementary Table S2.** Relative normalized expression values and the range of error based on the standard deviation for the dUTPase isoforms and the DUT-all target upon serum starvation and p-values as calculated by the CFX Maestro software.

| **Biological Group** | **Target** | **Non-treated** | | | **Serum starved** | | | **p-value** |
| --- | --- | --- | --- | --- | --- | --- | --- | --- |
|  |  | **Relative normalized expression** | **Exp. Lower Error Bar** | **Exp. Upper Error Bar** | **Relative normalized expression** | **Exp. Lower Error Bar** | **Exp. Upper Error Bar** |  |
| A-549 | DUT-M | 1 | 0.920 | 1.087 | 1.918 | 1.829 | 2.011 | 2.97E-04 |
|  | DUT-N | 1 | 0.881 | 1.136 | 0.261 | 0.219 | 0.311 | 4.28E-04 |
|  | DUT-3 | 1 | 0.823 | 1.215 | 3.928 | 3.694 | 4.176 | 3.17E-04 |
|  | DUT-4 | 1 | 0.828 | 1.208 | 1.154 | 0.910 | 1.462 | 4.60E-01 |
|  | DUT-all | 1 | 0.952 | 1.050 | 0.634 | 0.541 | 0.742 | 8.74E-03 |
| HeLa | DUT-M | 1 | 0.686 | 1.457 | 1.633 | 1.594 | 1.672 | 8.74E-02 |
|  | DUT-N | 1 | 0.814 | 1.229 | 0.900 | 0.874 | 0.926 | 4.30E-01 |
|  | DUT-3 | 1 | 0.684 | 1.463 | 1.888 | 1.855 | 1.922 | 4.45E-02 |
|  | DUT-4 | 1 | 0.864 | 1.158 | 1.085 | 0.914 | 1.287 | 5.64E-01 |
|  | DUT-all | 1 | 0.830 | 1.205 | 1.167 | 1.066 | 1.277 | 2.68E-01 |
| MDA-MB-231 | DUT-M | 1 | 0.932 | 1.073 | 2.210 | 1.520 | 3.213 | 2.26E-02 |
|  | DUT-N | 1 | 0.886 | 1.129 | 0.300 | 0.285 | 0.315 | 9.00E-05 |
|  | DUT-3 | 1 | 0.795 | 1.258 | 2.145 | 1.649 | 2.789 | 1.93E-02 |
|  | DUT-4 | 1 | 0.835 | 1.197 | 0.953 | 0.745 | 1.219 | 7.97E-01 |
|  | DUT-all | 1 | 0.879 | 1.137 | 0.350 | 0.339 | 0.361 | 1.64E-04 |
